# A scoping review of physical activity interventions to support the mental health of students aged 16 to 25

**DOI:** 10.1186/s12982-025-00933-8

**Published:** 2025-09-12

**Authors:** Andy Mahon, Caitlin Holt, Matthew Hibbert, Russell Jago, Laura Tinner, Judi Kidger

**Affiliations:** 1 Centre for Public Health, University of Bristol, Canynge Hall, Clifton, Bristol, BS8 2PL UK; 2 Somerset Council Public Health, County Hall, Taunton, Somerset, TA1 4DY UK

**Keywords:** Physical activity, Mental health, Young people, Intervention, Education, Student

## Abstract

**Background:**

The mental health of young people is a growing public health concern. While physical activity has both physical and mental health benefits, there is limited evidence on physical activity interventions to improve the mental health of young people aged 16 to 25. We aimed to scope the types and design of physical activity interventions in educational settings that target mental health outcomes for this group.

**Methods:**

A systematic search was conducted for articles published from 2015 to January 2025. References were screened and included for data extraction if they reported the effects of a physical activity-based intervention on mental health-related outcomes in students aged 16 to 25 in high income countries. Findings were reported according to PRISMA extension for scoping reviews.

**Results:**

A total of 22,437 records were identified and 51 studies met the inclusion criteria. Most were conducted in the USA (*n* = 16); just one study was conducted in the UK. Most were conducted in post-secondary settings like universities (*n* = 39) with less focus on older adolescents in upper secondary education (*n* = 12). Twenty studies were either RCTs or cluster-RCTs. Aerobic exercise (*n* = 26) and yoga (*n* = 15) were the most common physical activity interventions. Beneficial effects of the physical activity-based interventions were reported for the majority of the twenty-eight mental health outcomes assessed, notably depressive symptoms, anxiety and stress.

**Conclusions:**

There was considerable heterogeneity in design of interventions, components used and how mental health was measured. There was a lack of evidence on inequalities in outcomes including differences by gender or socioeconomic position. The evidence examined in this review suggests physical activity-based interventions within education settings could be an effective and feasible option to support a range of mental health outcomes in students aged 16 to 25. However, several under-researched areas were identified, including a need for more well-designed, high-quality studies to examine the mental health effects of physical activity interventions within older adolescent students, and examining the differences between genders and sub-groups of young people such as those from lower SES backgrounds or with learning or physical disabilities.

**Supplementary Information:**

The online version contains supplementary material available at 10.1186/s12982-025-00933-8.

## Introduction

The growing prevalence of mental health issues within older adolescents and young adults is a public health concern [1]. Globally, prevalence rates for mental health conditions in adolescents have been estimated at 14% [2]. This is higher in UK contexts with one in five young people aged 8 to 25 years in England experiencing mental health conditions; with slightly higher rates in the 17 to 19 years (23.3%) and 20 to 25 years (21.7%) age groups [1]. Students aged 16 to 25 face significant stressors that can affect their mental health, including transitions within education, academic pressure, increased independence, loneliness and financial stress [3]. In England, 95% of further education colleges report an increasing prevalence of mental health issues among their student populations [4]. Similarly, UK universities have seen significant increases in the rates of mental health issues amongst their students in recent years [5]. The same trend has been reported in other high-income countries such as the US, Canada and Australia [5, 6]. The period between 16 and 25 years is a developmentally important time in one’s life, and 75% of mental health disorders emerge by the age of 25 [7, 8]. Poor mental health in this age group can have lasting impacts on personal, social and academic development [9]. Ensuring that young people receive support for their mental health is important to minimise the risk of long-term consequences, however, increasing demand and stretched services often result in unmet needs among this age group [10, 11].

Education settings are key in the delivery of interventions [7]. Academic institutions may provide a more familiar and comfortable environment for young people in comparison to traditional clinical settings and have the potential to reach large numbers who may struggle to access mental health support [12, 13]. Many academic institutions such as schools and universities provide counselling or other individualised services [10]. However, with increasing demand, alternative and additional strategies to support the mental health of students and prevent problems developing is an important line of enquiry.

Physical activity (PA) has great potential for the improvement of mental health and could be effective and deliverable within education settings [14]. Regular PA is associated with both physical and mental health benefits, but many young people are not engaging in sufficient levels to achieve these benefits [15]. The World Health Organisation (WHO) guidelines recommend that children and adolescents should achieve an average of sixty minutes of moderate-to-vigorous physical activity each day, along with at least three vigorous intensity activities and activities that can strengthen muscle and bone per week [16]. For adults, it is recommended to engage in at least 150 to 300 min of moderate intensity activity, or 75 to 150 min of vigorous activity per week, along with muscle strengthening activities at least two times per week [15]. However, globally, nearly a third of adults and 80% of children and adolescents are not meeting these recommended amounts of weekly PA [17, 18]. In UK contexts, over half of children and young people are not meeting minimum daily levels [18–21]. PA typically declines through childhood and adolescence, and many young people disengage from sporting activities into adulthood [22, 23]. Around 80% of adolescents report their only source of physical activity comes from their education setting [24]. Thus, there is an opportunity to build upon the PA already taking place in education settings to deliver interventions that are effective for mental and physical health.

While the positive effects physical activity can have on mental health outcomes have been well-documented in the literature across demographics, the specific mechanisms through which these outcomes are achieved are not yet fully understood [25, 26]. A conceptual model has been proposed to describe the potential mechanisms responsible for the effects of physical activity on mental health in young people, which is comprised of three main mechanisms; neurobiological, psychosocial and behavioural [25]. Neurobiological mechanisms for the mental health and cognitive benefits of physical activity include structural and functional changes in the central nervous system through multiple pathways such as anti-inflammation, increased neuroplasticity, increased cerebral blood flow and blood vessel growth and neuroendocrine modulation [25, 26]. Psychosocial mechanisms suggested to contribute to the mental health impacts of physical activity include social outcomes like increased opportunity for social interaction and social support, connectedness and interaction with the outdoors and natural environment, along with psychological factors like increased self-esteem, self-efficacy, autonomy, purpose and self-perceptions [25]. Similarly, several behavioural mechanisms as a result of physical activity have also been suggested to play a role in the resulting mental health outcomes, such as increased sleep quantity and quality, less fatigue, and the development of behavioural skills such as self-regulation and coping skills that can positively impact an individual’s mental health [25, 26].

Despite the specific mechanisms not being fully understood, previous reviews have reported beneficial effects of physical activity-based interventions in young people, not limited to education settings, on mental health outcomes such as anxiety [27], depressive symptoms [27–29], negative affect [30], stress [30], self-esteem [27] and wellbeing. Just one previous study has reviewed the impact of PA interventions on mental health-related outcomes specifically within education settings [14]. The authors reported significant benefits on anxiety, resilience, wellbeing and positive mental health in children and young people of school-going age, 4 to 19 years. However, we have not identified a synthesis of the evidence for PA interventions to support mental health among the age group 16 to 25 within education settings. Therefore, scoping the contemporary evidence base for education setting-based PA interventions to support the mental health of older adolescent and young adult student populations is essential to inform future development of interventions.

While the contexts in which older adolescents and young adults attend education can differ significantly, for example between high schools and Universities, the overall aim of this review is to inform the future development and implementation of interventions within Further Education (FE) settings in England. FE is the education stage English students enter after secondary school, usually at age 16, encompassing the upper secondary education stage equivalent to high school [31]. English Colleges of FE cater for both older adolescent students of upper secondary education and adult learners of Higher Education within the same context. FE college settings are typically more reminiscent of third-level settings compared to secondary or high schools, due to features like increased independence and responsibility, and variety of courses and study modes; so findings from third-level settings are also meaningful and relevant to inform future FE-based interventions [32]. As we could identify no previous reviews which examined mental health outcomes of physical-activity interventions within 16–25 year old student populations, and due to the broad and exploratory nature of these study aims, we conducted a scoping review [33], with the following objectives:

1. To summarize the types of PA interventions that have been described in the literature to support the mental health of young people aged 16 to 25 in education settings in High-Income Countries.
2. To examine what mental health outcomes have been reported in the literature as a result of these PA interventions.
  - To summarize what evidence is available for the effectiveness of PA interventions in improving mental health outcomes.
  - To examine if differences or inequalities in mental health outcomes have been reported as a result of PA interventions for different subgroups of young people (e.g. gender, socioeconomic background).
3. To examine what barriers and facilitators to implementing PA interventions to support the mental health of young people aged 16 to 25 in education settings have been reported.
4. To identify gaps in the current literature on PA interventions to support the mental health of young people aged 16 to 25 in education settings in high-income countries.

## Methods

A systematic scoping review method was conducted following the 5-stage scoping review process [34, 35], and reported using the PRISMA guidance for Scoping Reviews [36].

**Stage 1: Identifying the research question and objectives**

The research questions were developed based on gaps in the evidence base and collaboration with stakeholders including public health professionals, college staff and young people.

**Stage 2: Identifying relevant studies**

The steps that were involved in identifying relevant studies for this review are outlined in Table 1. The inclusion criteria were based on the Joanna Briggs Institute guidance on Population, Concept, Context framework [37].

**Stage 3: Selection of studies**

Duplicate search results were removed using EndNote software. Titles and abstracts of search results were uploaded to the online software Rayyan (https://www.rayyan.ai) for screening. Two reviewers (AM & CH) independently screened 25% of titles and abstracts and discussed and resolved conflicts before one reviewer (AM) screened the remaining 75%. Two reviewers (AM & CH) then independently screened 25% of full text papers for eligibility, discussed and resolved conflicts and then one reviewer (AM) full-text-screened the remaining 75%.

**Stage 4: Data extraction**

A data extraction form was adapted to meet the aims of this review using the Template for Intervention Description and Replication (TiDIER) checklist and the JBI manual for evidence synthesis for scoping reviews [38, 39]. The extraction form was independently piloted by two reviewers (AM & CH) on 10% of included studies, with further changes made to enhance clarity and consistency of data extracted.

**Stage 5: Data synthesis**

A descriptive narrative summary of the PA interventions used and their effects on the mental health outcomes assessed is provided.

**Table 1:** Description of stage 2: identifying relevant studies of the scoping review methodology utilised within this review

Overview of stage 2: Identifying relevant studies	
**2.1 Inclusion criteria**	
*Population*	Studies were included if participants were students aged 16 to 25.
*Concept*	Studies were required to investigate the effects of PA interventions to support and improve mental health-related outcomes.
*Concept 1 – Physical activity interventions*	PA was defined as “any bodily movement produced by skeletal muscles that results in energy expenditure” [40]. Studies were included if they combined PA with another component, such as education or meditation, to map what types of intervention components achieved which outcomes.
*Concept 2 – Mental Health*	This review considered the whole continuum of mental health, from mental wellbeing to symptoms of mental health disorders such as anxiety and depression, to identify the range of mental health-related outcomes that have been researched. Studies of participants with eating disorders or that targeted eating disorder outcomes were excluded due to the complex relationship between PA and these disorders [41].
*Context*	Studies conducted in high income countries were included based on the World Bank’s 2023 Gross National Income (GNI) Per Capita High-Income classification [42]. The aim of the study is to inform future intervention development and implementation within a high-income context; contextual factors can have major impacts on both physical activity and mental health [43]. As such, to ensure relevance of findings to high-income contexts, studies in low or middle-income countries were excluded due to the contextual differences in education settings, lifestyle and cultural factors, and factors affecting the mental health of young people [44, 45].
	Studies were required to investigate interventions within a student population. Studies were not required to deliver the intervention exclusively within the education setting, as long as the population were full or part-time students, as many programmes may be offered in after-school timeslots or external locations. The International Standards for Classification of Education (ISCED) was used to describe and define education settings; Upper Secondary Education (ISCED level 3, e.g. high school) and Post-Secondary Education (ISCED level 4 – level 8, e.g. University [32].
**2.2 Types of sources**	This review included peer-reviewed, published studies of both experimental and quasi-experimental designs -such as randomised controlled trials (RCTs) and non-randomised controlled trials -and qualitative studies that met the inclusion criteria. Unpublished studies, grey literature and student theses were excluded. Protocols, opinion pieces, cross sectional and cohort studies and editorials were excluded. Systematic reviews were not included but the reference lists of reviews found during the search process were searched for relevant studies.
**2.3 Exclusion criteria**	• Studies published prior to 2015: This cutoff was chosen as the review was interested in recent, contemporary findings to ensure relevancy to the development and implementation of future interventions, and to prevent an unmanageable quantity of literature from being included that could have made the review logistically unmanageable and challenging to complete. • Studies conducted in low or middle-income countries as defined per World Bank [42]. • Studies published in a language other than English. • Studies that investigated a single or acute bout of PA and studies in which the intervention was shorter than 3 weeks were excluded as outcomes would be likely to be more transient [46].
**2.4 Search strategy**	A systematic search strategy was developed first by identifying relevant keywords with the aid of a thesaurus related to physical activity, mental health, education/students and interventions, along with identifying a list of relevant MeSH terms. Then with input from a subject librarian, the list of keywords and terms were expanded and developed into a search strategy. This was then piloted on the database Medline, further refined and then adapted for the further databases outlined below. The full list of keywords and search terms is presented as the Medline search strategy in Supplementary file 1. Searches were conducted in November 2023 for articles published between 2015 and 2023, and again in January 2025 for additional articles published between 2023–2025. Databases searched were: • Ovid Medline • Embase • PsychInfo • Educational Resources Information Center (ERIC) • British Education Index • Web of Science

## Results

### Sources

**Fig. 1:**
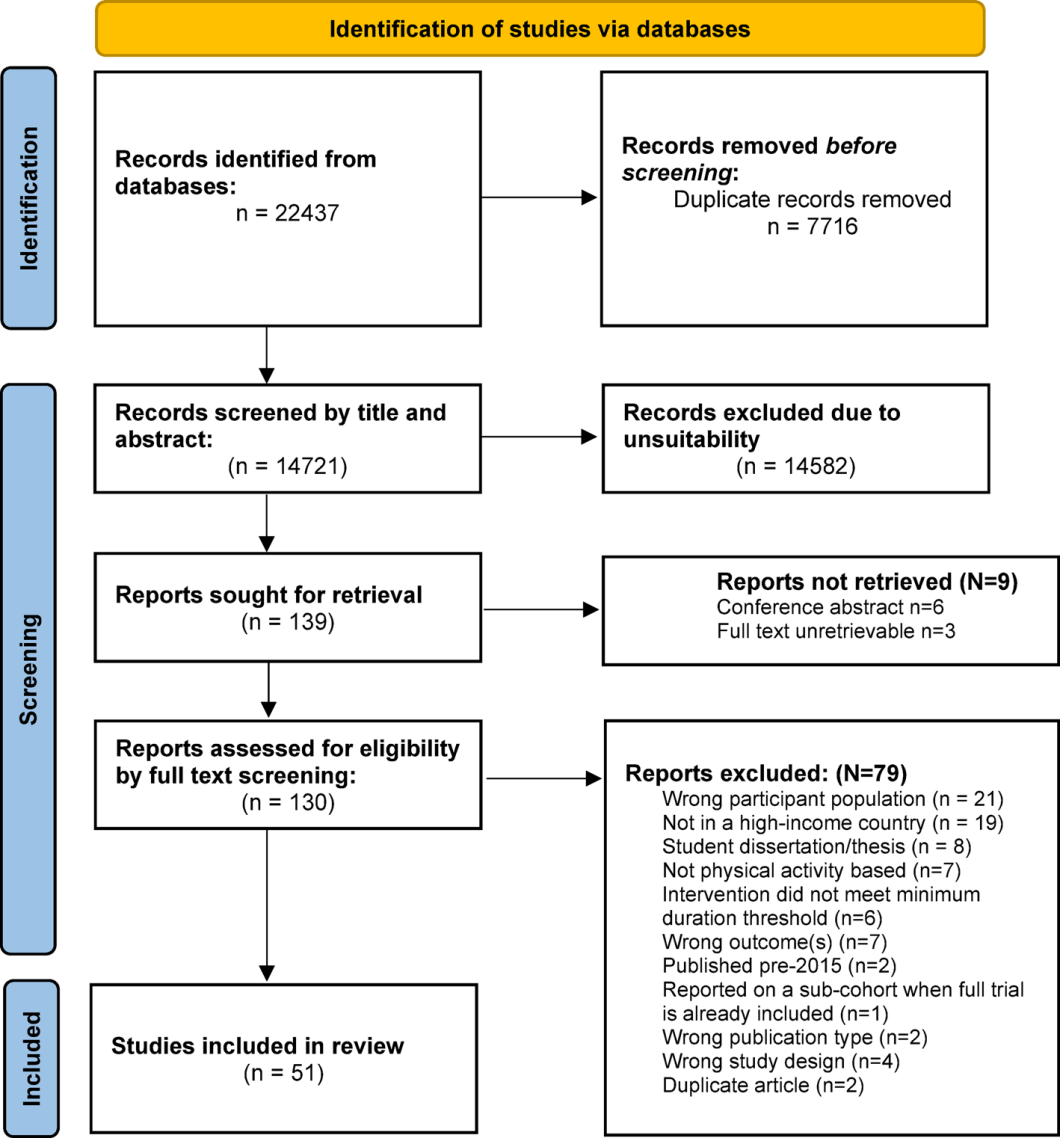
PRSIMA flow diagram presenting an overview of screening process. Searches were conducted in November 2023 for articles published between 2015 and November 2023, and then again in January 2025 for articles published between November 2023 to 22nd January 2025

In total, 22,437 results were identified in the search, and after removing duplicates and screening, 51 studies were included in this review. The PRISMA flow diagram including reasons for exclusion is presented in Fig. 1. Several of the included studies assessed two or more PA interventions, resulting in 57 interventions being reported in this review.

### Characteristics of included studies

An overview of the characteristics of included studies is presented in Table 2. The USA was the most common country of study delivery (*n* = 16), and most were published between 2020 and 2023 (*n* = 37). Most studies were conducted in post-secondary education settings (*n* = 39) with just twelve studies conducted in upper-secondary education settings.

### Quality of evidence

While a formal appraisal of the quality of the evidence was outside the scope of this review, the mixed quality of studies, and variety of study designs should be noted. A considerable amount of the studies were preliminary feasibility or pilot studies. Less than half of included studies were full-scale controlled trials, including RCTs (*n* = 18) and cluster-RCTs (*n* = 2).

**Fig. 2:**
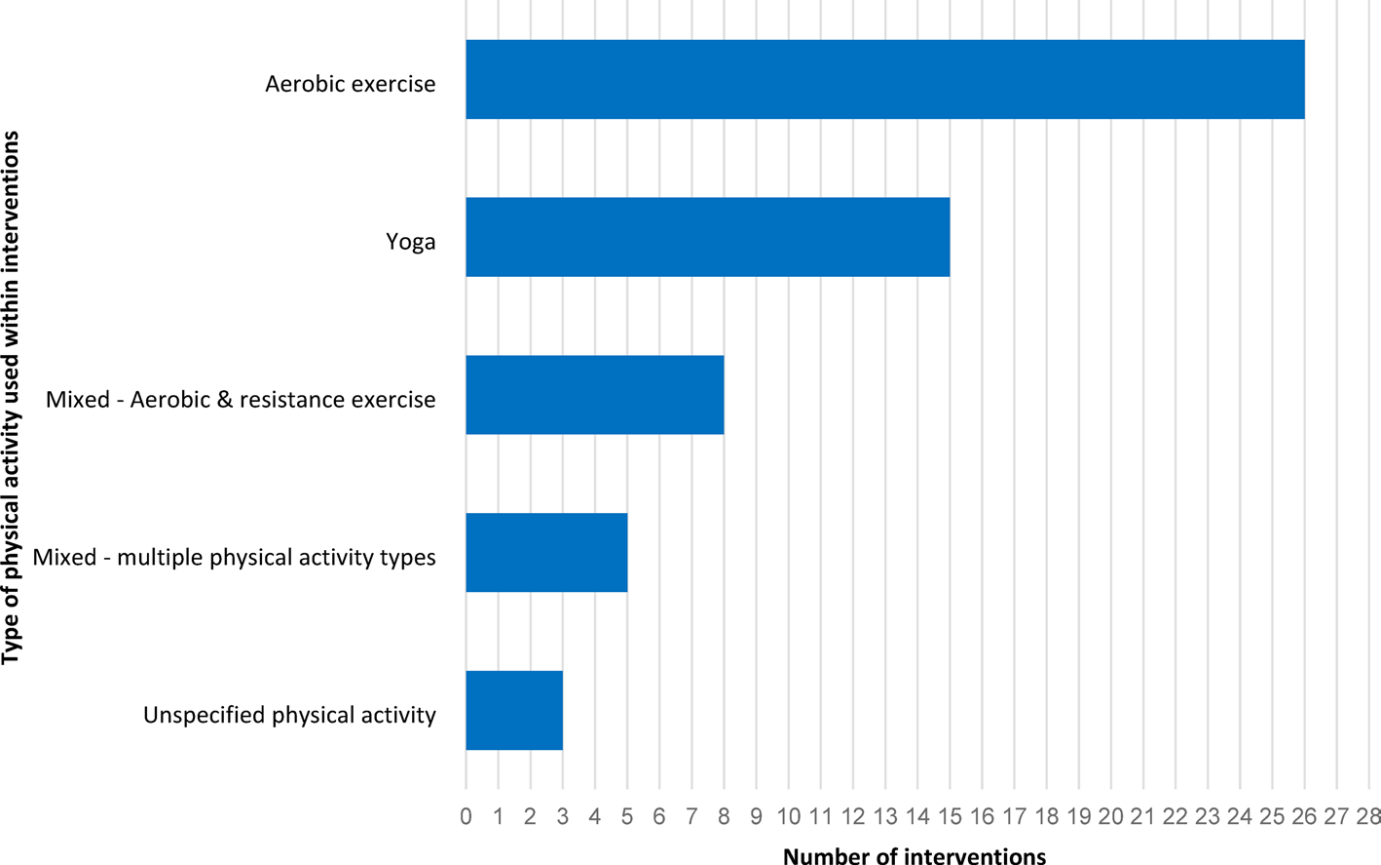
Distribution of types of physical activity utilized in the interventions reported. *N* = 57 interventions described in the 51 included studies

### Physical activity intervention characteristics

As presented in Fig. 2, the most common type of PA utilised within the 57 interventions was aerobic exercise (*n* = 26), followed by yoga (*n* = 15), mixed aerobic and resistance exercise (*n* = 8) and interventions combining multiple activity types (*n* = 5). Three studies did not state the specific type of PA used; these were individually tailored interventions in which participants or their trainers could select the type of physical activity, and were likely comprised of a variety of activities. Within interventions of aerobic exercise (*n* = 26), specific types included aerobic movements (*n* = 7), indoor exercise on a stationary bike or treadmill (*n* = 3), outdoor running (*n* = 5), walking or increased steps (*n* = 3), VR shadowboxing (*n* = 1) and dance (*n* = 1); six interventions did not specify the aerobic exercise used. All interventions were provided free of charge at no financial cost to the participants.

#### Intensity

Physical activity intensity was specified for 26 interventions including low intensity (*n* = 4) [46–50], low-to-moderate intensity (*n* = 2) [51, 52], moderate intensity (*n* = 6) [53–56], moderate-to-vigorous intensity (*n* = 6) [46, 57–61], and high intensity (*n* = 5) [55, 62–64]. Three interventions were comprised of mixed intensities; two interventions provided classes of multiple physical activity types and therefore likely a variety of different intensities [65, 66], and one intervention started off with running at low intensities and gradually increased the intensity as the intervention progressed [67]. Intensity was not specified for the remaining thirty-one interventions.

Beneficial effects of interventions of all of the above intensity categories were reported on mental health outcomes with no definitive pattern of evidence to support the recommendation of one physical activity intensity as optimal for achieving mental health benefits. However, interventions comprised of physical activity of moderate and moderate-to-vigorous intensities seemed to demonstrate more consistent beneficial effects on primary outcomes like depressive symptoms [55–57], stress and anxiety [46, 55].

#### Universal vs. targeted interventions

**Fig. 3:**
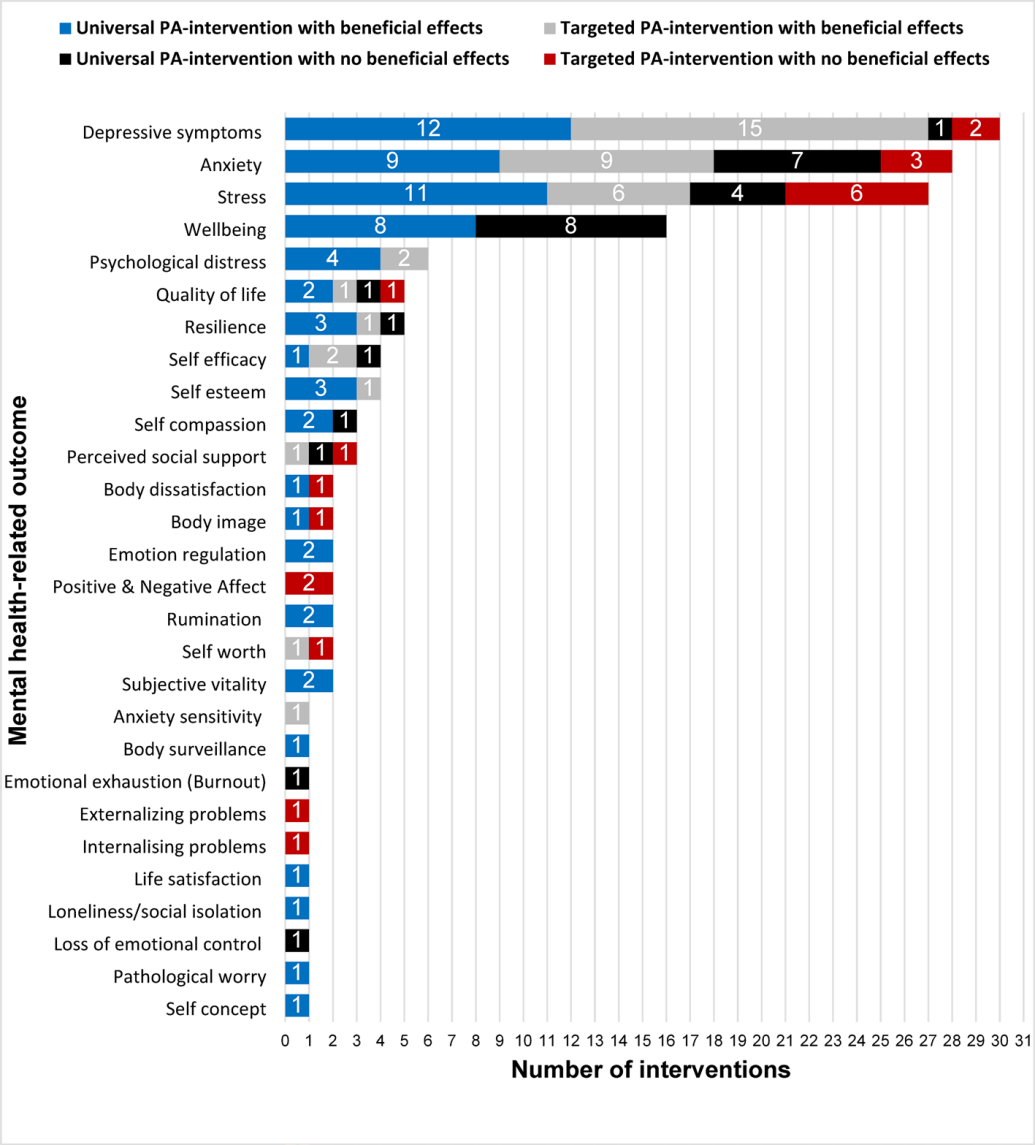
Distribution of mental health-related outcomes assessed within included studies by number of interventions examined, *n* = 57. Several studies assessed multiple outcomes, so total outcomes does not equal 57. Bars are stacked by the number of Universal interventions and Targeted interventions that demonstrated beneficial effects, and the number of universal and targeted interventions that had no beneficial effects on each mental health-related outcome. Targeted interventions refers to interventions in which participants where specifically recruited for a pre-identified feature, such as diagnosed or experiencing elevated mental health issues. While universal interventions refer to interventions open to participants from the general student population not targeted for a specific feature or diagnosis

The delivery of mental health interventions can be approached universally, where the intervention is offered to a whole population, or of relevance to this review, a whole school cohort for example, or in a targeted approach where individuals are selected based on already experiencing symptoms of or being at an increased risk of developing mental health issues [68, 69]. While most studies investigated interventions that took a universal approach (*n* = 32, examining 36 different interventions), the remainder took a targeted approach (*n* = 19, examining 21 different interventions). Criteria for recruitment within targeted interventions included experiencing baseline mental health issues such as depression, anxiety, psychological distress or high levels of mental fatigue (*n* = 15), being inactive defined as engaging in less activity than guideline daily PA amounts (*n* = 2) or overweight/obese (*n* = 1) and being characterized as ‘at-risk’ youth (*n* = 1). In comparison to universal interventions, interventions targeting participants with elevated mental health issues at baseline reported more consistent beneficial effects on outcomes such as depressive symptoms (15 of 15 interventions), stress (6 of 6), anxiety (8 of 9). A comparison of the number of universal and targeted interventions that had beneficial effects on mental health-related outcomes is depicted in Fig. 3.

#### Delivery setting, mode and personnel

The majority of interventions (*n* = 44 of 57) were delivered to students of post-secondary settings. Of the 57 interventions, the majority were comprised of supervised sessions (*n* = 42), delivered either in groups (*n* = 35) or one-on-one (*n* = 7). Most supervised interventions were delivered in-person within the education setting such as in the classroom or campus facilities (*n* = 30) or external facilities such as local fitness centres or local yoga studio (*n* = 3); three did not specify the in-person location. The remainder of supervised interventions were delivered virtually via video conferencing platforms (*n* = 6). Unsupervised interventions (*n* = 15) included independent unsupervised PA (*n* = 9), pre-recorded video guides participants could follow along to (*n* = 4) and independently conducted walking or increased step interventions (*n* = 2). Of the supervised interventions, sessions were delivered by qualified instructors of the relevant PA type (*n* = 22), researcher(s) (*n* = 8), other students who were studying a physical activity-related specialisation (*n* = 3), teachers (*n* = 3), a multi-disciplinary team including mental health professionals and physical trainers (*n* = 3) and three did not specify who delivered the sessions. Interventions delivered by qualified instructors of the relevant type of PA demonstrated more consistent beneficial effects in comparison to other delivery personnel, notably on depressive symptoms (11 of 13 interventions), stress (10 of 12 interventions), anxiety (10 of 11 interventions) and wellbeing (5 of 6 interventions). However, this may be due to the considerably higher number of interventions delivered by qualified instructors in comparison to the other noted personnel. Both supervised and unsupervised interventions demonstrated beneficial effects on mental health outcomes.

#### Intervention duration, session duration and session frequency

The duration of interventions varied, ranging from 3 weeks to 24 weeks. Most lasted between 6 and 8 weeks (*n* = 35). The duration of sessions within interventions ranged from five minutes to 90 min, including sessions of less than 30 min (*n* = 14), 30 to 45 min (*n* = 11), 45 to 60 min (*n* = 13) and more than 60 min (*n* = 13). Seven interventions did not specify the duration of sessions. Most interventions consisted of three (*n* = 18), two (*n* = 17) or one (*n* = 12) sessions per week. Total weekly PA time accumulated within interventions ranged from 10 to 210 min, including less than 30 min per week (*n* = 5), 30 to 60 min (*n* = 18), 61 to 90 min (*n* = 10), 91 to 149 min (*n* = 5) and 150 min or more (*n* = 12); the total weekly PA time accumulated was unable to be extracted for the seven interventions that did not provide the duration of PA sessions per week.

There was no discernible pattern of effectiveness of interventions based on duration of sessions or total weekly minutes of PA provided by interventions. For outcomes such as depressive symptoms, anxiety, stress and wellbeing, beneficial effects were reported for interventions providing as low as 24 min [52] to as high as 210 min [53] of weekly physical activity.

### Mental health outcomes

There were twenty-eight distinct mental health-related outcomes examined in the included studies. As presented in Fig. 3, depressive symptoms was the most examined outcome. A narrative summary is provided below.

#### Depressive symptoms

The effects of thirty different interventions were examined on depressive symptoms; the most commonly assessed outcome (*n* = 24 studies, examining 30 distinct interventions). Study design varied; six studies were RCT’s [50, 55, 57, 70–72], the remaining were pilot studies (*n* = 7) [52, 56, 64, 67, 73–77], quasi-experimental (*n* = 6) [51, 54, 60, 78–80], and feasibility studies (*n* = 3) [6, 81, 82]. Self-report measures were used in all the studies; eleven different measures were used, the most common being the Depression Anxiety Stress Scales (DASS-21) (*n* = 6).

Of the 16 aerobic-PA-based interventions that examined depressive symptoms, a majority (*n* = 13) demonstrated beneficial effects, including interventions comprised of unspecified aerobic exercise (*n* = 4) [57], indoor stationary cycling or running (*n* = 3) [55, 56], outdoor running (*n* = 4) [50, 64, 67, 77], dance (*n* = 1) [71] and brisk walking (*n* = 1) [78]. Eight of these aerobic interventions reported effect sizes, which ranged from medium, Cohen’s d = 0.62 [52] to large, Cohen’s d = 1.88 [71]. Seven out of 9 yoga interventions had beneficial effects on depressive symptoms [51, 60, 70, 75, 76, 81]. Effect size was reported in two of these; Cohen’s d = 0.36 [81] and d = 1.04. Beneficial effects on depressive symptoms were reported for each of the mixed aerobic and resistance exercise interventions that looked at this (*n* = 3) [6, 60, 79], two of which reported effect sizes; Cohen’s d = 0.36 [79] and d = 0.67 [6]. Two individually tailored interventions of mixed activity types (*n* = 1) [82] and unspecified self-selected PA (*n* = 1) [74] had beneficial effects on depressive symptoms, with a medium effect size, d = 0.78 [82] and large effect size, η²= 0.43 respectively [74].

#### Anxiety

Anxiety was examined as an outcome in 23 studies, within which the effects of 28 different interventions were reported. Anxiety was measured using self-report questionnaires in all studies; eleven different measures were used, the Beck Anxiety Inventory being the most commonly used (*n* = 6) [83]. Quality of evidence was mixed; studies were randomized controlled trials [50, 55, 62, 70, 72, 84], along with pilot studies [52, 56, 64, 67, 73, 75–77, 85–87], studies with a quasi-experimental design [51, 60, 78–80] and a feasibility study [6].

Eight of the twelve aerobic-based interventions had beneficial effects on anxiety, including interventions consisting of outdoor running (*n* = 2) [67, 77], indoor stationary cycling/treadmill running (*n* = 2) [55, 56], unspecified aerobic exercise (*n* = 2) [64] and walking (*n* = 2) [78, 80]. Effect sizes were reported for two of these interventions and both reported large effect sizes, Cohen’s d = 0.97 [56]; Cohen’s d = 0.91 [55]. Five aerobic exercise interventions had no effect on anxiety including interventions consisting of outdoor running [50], virtual reality shadowboxing (*n* = 1) [86], aerobic movements [52, 86] and a high-intensity interval training (HIIT) stationary cycling intervention [55]. Two of the four interventions consisting of aerobic and resistance exercise reported beneficial effects on anxiety; both were individually tailored interventions for university students referred from campus mental health services and reported medium effect sizes [6, 79]. A remotely-delivered Zoom-based aerobic and resistance PA intervention and a HIIT-based aerobic and resistance intervention had no effect on anxiety (Cohen’s d = 0.02) [62]. Eight of the eleven yoga-based interventions had beneficial effects on anxiety [51, 70, 72, 73, 75, 84, 85, 87], two of which reported effect sizes; one reported a small effect size for state anxiety (Cohen’s d = 0.38) and medium effect size for trait anxiety (Cohen’s d = 0.8) [84], while the other reported a large effect size, Cohen’s d = 1.11 [51].

#### Stress

Stress was an examined outcome in 24 studies, including 27 interventions; *n* = 23 used self-report measures of stress, while one used a self-report measure alongside hair cortisol concentrations as a physiological measure [63]. Eight different self-report measures were used, the most common being the Perceived Stress Scale (*n* = 13) [88]. Again, study design varied; studies were RCTs (*n* = 9) [46, 50, 55, 62, 70–72, 84, 89] or cluster RCTs (*n* = 1) [25], quasi-experimental (*n* = 4) [49, 51, 54, 66], pilot studies (*n* = 9) [52, 56, 67, 73, 75, 85–87, 90] or feasibility studies (*n* = 1) [81].

Eight of the eleven aerobic-PA interventions had beneficial effects on stress, including interventions comprised of indoor stationary cycling or treadmill running (*n* = 2) [55, 56], unspecified aerobic exercise (*n* = 2) [52, 54], outdoor running (*n* = 2) [50, 67], VR shadowboxing (*n* = 1) [86] and dance (*n* = 1) [71]. Effect size was reported for three of these interventions, ranging from medium (Cohen’s d = 0.72) to large (Cohen’s d = 1.24) [52, 56, 71]. Almost all of the yoga-based interventions demonstrated beneficial effects on stress (*n* = 8 of 9) [51, 70, 72, 73, 81, 84, 85, 87], with effect size reported in three of these (Cohen’s d = 0.44, 0.49 and 1.34) [51, 81, 84]. Of the five interventions comprised of aerobic and resistance training, one intervention of moderate to vigorous intensity aerobic and resistance exercise sessions had beneficial effects on self-report stress [46], while the ‘Burn2Learn’ HIIT intervention reduced hair cortisol concentrations in the intervention group, but had no effects on the self-report stress measure [63]. The other three aerobic and resistance PA interventions did not report any effects on stress [49, 62, 89]. Three others examined the effects of interventions based on mixed activities on stress as an outcome [66]. One intervention comprised of Cognitive Behavioral Therapy (CBT) and mixed activity classes had marginal benefits on stress, however these effects did not differ from the control [46, 66, 90]. The two others combined light movement activities like relaxed, light-intensity yoga and gentle stretching with aerobic PA sessions, one of which reported beneficial effects [46].

#### Wellbeing

Thirteen studies reported on the effects of sixteen different interventions on wellbeing. Wellbeing was measured using seven different self-report scales, the most common being the World Health Organization 5-Item Subjective Wellbeing Index (WHO-5) (*n* = 4) [91]. Study designs varied, including RCTs [53, 84, 92–94] or cluster RCTs [63], pilot studies [52, 73, 90, 95], quasi-experimental [49, 58] and feasibility studies [65].

Of the eight aerobic exercise-based interventions, a digitally-delivered intervention of short, guided aerobic-exercise videos [94] along with three interventions of unspecified aerobic exercise had beneficial effects on wellbeing [53]. Of the four yoga-based interventions, three had beneficial effects on wellbeing; two of which reported small effect sizes, Cohen’s d = 0.36 and 0.47 respectively [84, 92]. Three interventions consisted of aerobic and resistance exercise, none of which had effects on wellbeing [49, 58, 63]. One intervention comprised of mixed activity types such as yoga, Pilates, resistance training and aerobic exercise had beneficial effects on wellbeing but did not report effect size [65], while another combining yoga, stretching, movement and walking had no effect [90].

#### Psychological distress

Five studies examined the effects of seven interventions on psychological distress [6, 46, 92, 94, 96]. Four different self-report measures were used to assess psychological distress, the most common being the Kessler Psychological Distress Scale (*n* = 2) [97]. Studies were RCTs [46, 92, 94], a feasibility study [6] and a study with a quasi-experimental design [96].

Beneficial effects on psychological distress were reported for all six interventions. An individually tailored intervention comprised of aerobic and resistance exercise decreased psychological distress with a large effect size, Cohen’s d = 0.78 [6]. A yoga-based intervention decreased psychological distress at post-intervention and six month follow up with a small effect size, Cohen’s d = 0.32 [92]. An individually tailored intervention decreased psychological distress among at risk older adolescents with a large effect size, Cohen’s d = 1.03 [96]. While not statistically significant, there were improvements in psychological distress after both a light intensity intervention combining yoga-like stretching movements and outdoor walking, and a moderate-to-vigorous intensity intervention of aerobic and resistance training (Cohen’s d = 0.54 and 0.38) [46]. A remotely delivered intervention of daily self-guided, short duration aerobic-exercise videos had a beneficial effect, however no effect size was reported [94].

#### Quality of life (QoL)

Five studies including RCTs (*n* = 2) [93, 94], a feasibility study [82], a pilot study [75] and a quasi-experimental study [54], examined the effects of five interventions on QOL. Beneficial effects were demonstrated for three of the interventions. The STRIDE individually tailored exercise intervention had beneficial effects on QOL with a large effect size on the mental health subscale of the SF12, Cohen’s d = 1.04; although there was no effect on the physical health subscale, Cohen’s d = 0.06 [82]. There was an improvement in QOL after the ‘MAP: Train my brain’ meditation and aerobic exercise intervention [54], and after a once-weekly yoga intervention [75]; neither reported effect sizes. There were no effects on QOL of a pedometer-based intervention to increase student’s steps to 10,000 per day [93], or of a remotely-delivered intervention of daily, guided aerobic-exercise videos [94].

#### Resilience

Three studies examined the effects of five interventions on resilience, including two studies of a quasi-experimental design [58, 96] and a pilot study [76]. Four of the five interventions reported beneficial effects. Two remotely delivered group interventions, one a mindfulness-based yoga intervention and the other a moderate-to-vigorous aerobic and resistance exercise based intervention, reported beneficial effects on resilience; however, effect sizes were not reported [58]. An individually tailored intervention for at-risk adolescents, comprised of online health coaching and coached physical activity, had beneficial effects on resilience with a large effect size, Cohen’s d = 1.88 [96]. While neither of two trauma-based yoga interventions, one delivered face-to-face and one delivered remotely, had statistically significant effects, the face-to-face intervention had meaningful improvements in resilience [76].

#### Self-efficacy, self-esteem, self-compassion, self-worth, and self-concept

Four studies investigated the effects of PA-interventions on self-efficacy; two RCT’s [47, 48, 61], a feasibility study [82] and a quasi-experimental study [80]. Three reported beneficial effects of the interventions, with two reporting large effects sizes; a moderate-to-vigorous aerobic exercise intervention (Cohen’s d = 2.34) [61], and an individually tailored exercise intervention for students with a mental health diagnosis (Cohen’s d = 1.19) [82]. An intervention delivered through a digital health management application in which participants were instructed to walk for 30-mins per day along with incentivizing challenges and health education materials had beneficial effects, but did not report an effect size [80]. An RCT reported no significant effects on self-efficacy in university students after a 6-week low intensity running intervention [47, 48].

Two studies examined the effects of four interventions on self-esteem. An RCT reported beneficial effects on self-esteem of three moderate-intensity aerobic exercise interventions [53]. An individualized intervention in which sedentary adolescent females were provided with free access to a local fitness centre and encouraged to engage in PA at least once per week reported significant improvements in self-esteem, which were maintained at one-year follow up; no effect size reported [98].

Two studies examined the effects of physical activity interventions on self-worth [98, 99]. A pilot study reported small, non-significant benefits on self-worth in the full cohort after an intervention supplementing PE classes with yoga sessions compared to PE as usual for older adolescent high school students in the USA [99]. However, as there was only one male participant, when the authors ran the analysis with just female participants, there was a significant moderate increase in self-worth after the yoga intervention compared to PE as usual. An RCT reported beneficial effects on self-worth after a 24-week targeted intervention on self-worth in older adolescent female high school students in Sweden [98].

One RCT reported no effects on self-compassion of an 8-week yoga intervention in University students with a diagnosis of depression or anxiety; interestingly, the time matched mindfulness comparator intervention did result in a significant increase in self-compassion [70]. A quasi-experimental study reported significant positive effects of an 8-week low-to-moderate intensity yoga intervention in University students with a large effect size, Cohen’s d = 1.15 [51]. Another RCT reported significant positive effects of a 6-week yoga intervention on self-compassion of Portuguese university students [72]. However, the authors reported no significant effects of the intervention on self-concept [72]. One quasi-experimental study did report significant positive impacts of a 12-week daily brisk walking intervention on self-concept in adolescent high school students in Taiwan [78].

#### Body image, body dissatisfaction and body surveillance

Two studies investigated body dissatisfaction as an outcome [52, 66]. An aerobic-exercise based intervention resulted in a non-significant reduction in body dissatisfaction [52], while a ‘Body and Mind’ CBT and exercise intervention comprised of classes of multiple PA types decreased body dissatisfaction with a small to medium effect size, Cohen’s d= −0.4 [66].

Two studies investigated body image [66, 99]. There was significant improvements in positive body image measured by the BAS-2 (Cohen’s d = 0.7), and the appearance evaluation (Cohen’s d = 0.5) and body satisfaction (Cohen’s d = 0.7) subscales of the MBSRQ-AS, while there was reductions in negative body image constructs, including body image concerns measured using the SATAQ-4 scores (Cohen’s d = 0.6), and appearance orientation (d = 0.9) measured using the MBSRQ-AS compared to a waitlist control after the ‘Body and Mind’ CBT and PA-intervention [66]. However, a 12-week intervention supplementing PE class with yoga sessions found no difference in effects on body image compared to PE as usual [99]. The authors did report, however, positive effects of the intervention on body surveillance, with a moderate decrease compared to the PE as usual control (*p* < 0.05, η²ρ = 0.19).

#### Other mental health outcomes

##### Anxiety sensitivity

One study reported significant beneficial effects on anxiety sensitivity in female students with high anxiety sensitivity at baseline after a 14-week cognitive behavioural therapy and aerobic exercise intervention; however, there was no significant effect in female students with low anxiety sensitivity at baseline [50].

##### Emotion regulation

An RCT examined the effects of the ‘Bent on Learning’ 16-week yoga intervention on emotion regulation measured using the ERICA scale [100]. There was a significant beneficial effect on emotion regulation reported for the intervention group compared to PE as usual control group, effect size not reported [100]. Another RCT reported no significant effects of a 6-week yoga intervention on emotion regulation in Portuguese university students [72].

##### Emotional exhaustion

An RCT reported no significant effects on emotional exhaustion (Burnout) of a 6-week low intensity running intervention in Dutch university students [47, 48].

##### Externalizing and internalizing problems

A cluster RCT reported no significant effects on externalizing problems or internalizing problems after the ‘Burn 2 Learn’ HIIT intervention in Australian older adolescent secondary school students [63].

##### Life satisfaction

An RCT reported significant positive effects on life satisfaction after a 12-week yoga intervention (Cohen’s d = 0.36) [92]. The increase was maintained until 6 month follow-up (Cohen’s d = 0.22).

##### Loneliness and social isolation

A feasibility study reported significant positive effects on loneliness and social isolation after a 6-week intervention comprised of a variety of physical activity classes such as yoga, Pilates and HIIT, along with short health education workshops at the beginning of sessions in Irish medical school students [65].

##### Loss of emotional control

Loss of Emotional Control scores were decreased after a 6-week individualized exercise intervention for Canadian University students with a diagnosis of depression or anxiety [79]. While not significant, loss of emotional control subscale scores decreased by a mean 2.18 (95% CI, − 0.02 to 4.39).

##### Pathological worry

There was a significant reduction in participant Pathological worry scores (*p* = 0.001, ηp² = 0.67) after a 6-week yoga intervention for American university students with elevated levels of stress and/or anxiety [87].

##### Perceived social support

Perceived social support was examined in three studies [65, 80, 96]. One study reported significant positive effects of a personalised exercise intervention for adolescents categorised as ‘at-risk’ youth vocational secondary schools of low SES-backgrounds in Israel, with a large effect size (Cohen’s d = 1.11) [96]. However, there was no significant effects on perceived social support after a 6-week physical activity and health education intervention in Irish medical school students [65] or a 12-week digitally-delivered walking intervention for Korean University students [80].

##### Positive and negative affect

Two studies examined the effects of PA interventions on positive and negative affect; both used the Positive and Negative Affect Schedule (PANAS) self-report measure. Negative affect was decreased after an aerobic and resistance exercise intervention in University students, although this decrease was also reported for the non-PA comparator (expressive writing) intervention group [49]. Positive affect decreased and negative affect increased between pre and post after an aerobic exercise intervention [52].

##### Rumination

One study reported significant reductions in rumination amongst university students compared to control after the 8-week ‘MAP: train my brain’ intervention, which combined meditation and moderate intensity aerobic exercise [54]. Overall ruminative thoughts were reduced by 17% and depressive rumination and brooding rumination subscales were reduced by 16% and 24% respectively after the PA-intervention, with no changes in the control. Another pilot study reported significant positive effects on rumination after a 6 week yoga intervention for university students with elevated stress and/or anxiety [87].

##### Subjective vitality

The effects of two interventions on subjective vitality, measured using the Subjective Vitality Scale, in U.S university students were examined in a quasi-experimental study [58, 59]. ‘WeActive’ was an aerobic and resistance training intervention, and ‘WeMindful’ was a mindfulness-based yoga intervention. Both interventions comprised of 2 × 30-min sessions of their respective PA-type per week for 8 weeks, delivered virtually through ZOOM. Both the WeActive and WeMindful interventions had beneficial effects on subjective vitality (η²= 0.088), although the WeMindful intervention had more prominent increases in subjective vitality.

### Impact on inequalities

#### Gender

Four studies recruited female-only samples, which reported beneficial effects on positive body image, body dissatisfaction, self-worth, self-esteem and self-efficacy [50, 61, 66, 98]. One study recruited a very low number of males due to an imbalance in gender distribution in the recruitment setting and ran the analysis with a female only sample, which reported stronger beneficial effects on self-worth with the female-only cohort [99].

Two studies reported inequalities in outcomes based on gender; both of which examined yoga-based interventions in upper secondary education settings. Firstly, Jeitler and colleagues [73], reported a 12-week yoga intervention had more prominent effects on anxiety, depressive symptoms and stress in female participants than their male counterparts. In a companion qualitative evaluation, Jeitler et al. [101] reported that males also rated yoga as less beneficial and school sports more beneficial, while the opposite was true for female colleagues.

There were no significant effects of a yoga intervention on self-worth in a full mixed sample, however, gender was identified as a significant covariate [99]. When analyses were run on a female only sample with males excluded, there was a significant beneficial effect on self-worth.

#### Socioeconomic factors

While the link between low socioeconomic status (SES) and risk of poor mental health has been identified previously [102]; the relationship between low SES and low levels of PA has been suggested but is still uncertain [103, 104]. However, understanding how these factors relate to the delivery and impact of PA interventions to support the mental health of students is important to minimise intervention-generated health inequalities. Just four studies included in this review reported participants’ socioeconomic information [62, 63, 78, 100]. One study specifically recruited ‘at-risk’ youth who were attending vocational schools typically of low SES backgrounds, which reported beneficial effects on psychological distress, resilience and perceived social support, all with large effect sizes, Cohen’s d > 1 [96]. No study reported an analysis of outcomes based on SES. None of the included studies investigated the effects of physical activity interventions on mental health outcomes in young people with learning or physical disabilities.

### Barriers, facilitators and feasibility of PA-interventions in the education setting

Eight studies provided an evaluation of the feasibility and acceptability of interventions, each of which reported PA interventions were deemed acceptable and appropriate by students [6, 59, 62, 63, 65, 73, 82, 101]. Participant satisfaction was rated as high [59, 62, 73, 82, 101] or moderate-to-high [63], while just one study reported teacher satisfaction as high for a HIIT intervention in an upper secondary education setting [63]. Two studies reported adverse effects of the interventions, including unspecified injuries occurring due to the intervention, causing the participant(s) to drop out [48], and mild, short-term side effects such as headache and joint pain immediately after yoga sessions [73]. Sixteen studies reported participant attendance rates of sessions within interventions. While one study reported a 100% attendance rate [55], most ranged from 52 to 70% attendance, and eight studies reported low attendance rates as a primary factor that negatively impacted the delivery of the intervention.

Implementation barriers included scheduling issues, such as the timing of the PA sessions or conflicts with the academic schedule (*n* = 6 studies) [62, 65, 66, 71, 81, 85], followed by a perceived lack of time among participants (*n* = 4) [54, 56, 74, 98] and high academic pressure, such as too much school work or exam pressure (*n* = 2) [70, 98]. In one study, students reported that low confidence to engage in PA was a barrier, but cited the individual, one-on-one nature of the intervention and private exercise location on campus as factors that helped overcome this [6].

Four factors that enhanced the implementation of the intervention or participants’ ability to engage were reported. These included using technology to enhance flexibility in the facilitation of the intervention, such as delivering sessions virtually or providing pre-recorded materials [52, 58, 59, 96], implementing strategies to increase attendance, such as offering course credits for participation or making participation mandatory as part of a curriculum [57], use of multiple PA types to be more accessible to varying levels of fitness and to cater for variable preferences of participants [65] and the use of behavior change strategies such as goal setting [6, 98].

## Discussion

This scoping review examined the available evidence for physical activity-based interventions to support the mental health of students aged 16 to 25. Fifty-one studies were included which investigated fifty-seven different physical activity-based interventions. The majority of studies were conducted within post-secondary education settings. Broadly, the evidence suggests PA could be an effective intervention option to support the mental health of older adolescent and young adult students.

### Physical activity interventions

There was considerable variation in the characteristics of the fifty-seven PA interventions within the included studies. Types of PA utilized included multiple forms of aerobic exercise, yoga, mixed aerobic and resistance training and combining multiple exercise classes such as Pilates and HIIT. Consistent with previous research, the most commonly utilised type of PA within interventions was aerobic exercise [105]. Unsurprisingly, aerobic exercise-based interventions had the strongest weight of evidence, particularly in respect to outcomes such as depressive symptoms, stress and anxiety. These interventions varied considerably in terms of intensities, durations and specific forms of aerobic exercise used, including indoor cycling, running, aerobic movements and dance.

A relatively high number of interventions were based on yoga. The potential for yoga as an intervention to support mental health in the school setting has been suggested previously [106, 107]. This review provides further evidence indicating the potential effectiveness of yoga as a mental health promotion strategy within upper and post-secondary education contexts, particularly on perceived stress. Academic stress is consistently cited as an important factor affecting the mental health of students, and yoga could be an effective stress-mitigation option for education providers [108]. However, while the majority of these interventions were delivered within the education setting, almost all were delivered by external, certified yoga instructors, which could make yoga interventions less universally feasible for academic institutions if this increases the delivery costs and resources required.

Resources could also explain why there was less of a focus on other common forms of PA such as resistance training, which was a component of just nine interventions reported in this review. Resistance exercise interventions requiring expensive equipment, space and appropriate supervision could be costly and more difficult to deliver [109]. However, most of the interventions including resistance-based exercise reported in this review used bodyweight movements, eliminating the need for equipment and reducing cost; making it a potential option for future interventions. Previous research has shown the beneficial effects of resistance training on mental health outcomes in adult populations, particularly on depression and anxiety [110, 111]. This was also the case with interventions identified in this review; of the six interventions including resistance training components, three reported beneficial effects on depressive symptoms and anxiety, with a further one intervention each reporting beneficial effects on psychological distress, stress and resilience. However, no beneficial effects were reported on other primary outcomes such as wellbeing and stress. Furthermore, just two of these interventions was conducted with adolescents in upper secondary settings. While this evidence suggests interventions including resistance training could have beneficial effects on student mental health, the comparative lack of research particularly within older adolescent students warrants more future research.

Overall, the evidence from this review suggests a variety of types of PA can be beneficial for the mental health of students. However, the heterogeneity in intervention characteristics makes it difficult to draw any meaningful conclusions as to which type of PA may be the most effective for mental health outcomes [14, 112]. It has been suggested that the type of PA may be less important than other factors such as context, duration or frequency [113]. For example, previous recommendations suggest a minimum duration of 10 weeks for physical activity-based interventions for adults with depression [105]. Most interventions included in this review were of a shorter duration, lasting between four and eight weeks, which was flagged as a limitation in several studies [48, 81]. Students are typically enrolled for at least an academic year, making it potentially feasible to deliver longer term interventions, which is a recommendation for future investigation.

While most interventions provided between one and three sessions a week, the duration of these sessions ranged from five to ninety minutes in length. It has been suggested that mental health benefits can be gained through even small quantities of physical activity, which was evident with depressive symptoms in the studies included in this review [105]. Beneficial effects were reported for interventions accumulating as low as 24 min of weekly physical activity [52]. However, a pattern emerged whereby the interventions that had a low dose of PA per session, and lower total weekly quantity of minutes engaged in PA, did not have beneficial effects on stress [49, 62, 63], anxiety [50, 52, 62] or wellbeing [49, 52, 63]. For these outcomes, it may be necessary to provide a higher quantity of PA in terms of length of sessions and total quantity of sessions per week. Future interventions should acknowledge identified barriers, such as perceived lack of time and conflicts with academic schedules, when deciding frequency and session duration, along with the mode of delivery of sessions, such as online or in-person. Balancing these factors is important to ensure interventions do not cause unintended stress on time-poor students and to facilitate optimal uptake.

### Mental health outcomes

Beneficial effects of PA interventions were reported for twenty-four of the twenty-eight reported mental health-related outcomes, particularly depressive symptoms, stress and anxiety. Over half of the identified outcomes were examined in just one or two studies, so further research is necessary before any conclusions can be drawn as to the effectiveness of PA interventions for these outcomes.

There is a debate as to which intervention approach is best between universal and targeted interventions, where individuals are recruited to an intervention based on a diagnosis or baseline level of mental health issues; with some evidence to suggest targeted interventions may be more effective for the mental health of young people [114, 115]. The findings of this review suggest both targeted and universal PA-based interventions could be effective for a range of mental health outcomes in older adolescent and young adult students. For instance, there was a larger quantity of evidence in favour of targeted interventions for depressive symptoms and anxiety. This chimes with a recent study that reported that of the 36% of UK Universities that offer PA programmes, the majority of these (88.9%) are targeted, referral-based PA interventions [5]. Given most academic institutions have some form of student support or pastoral care service, PA could be an effective referral-based, individualised treatment option for young people in upper or post-secondary education settings. The individualised nature of these interventions may limit the reach and feasibility within education settings. Universal PA-interventions for full-student populations could facilitate increased reach across a wider range of students in comparison to targeted interventions and could be part of a prevention (rather than treatment) strategy for mental health in education settings. The evidence from this review suggests universal PA-interventions can be effective for a range of outcomes related to student mental health, notably stress and wellbeing, so it is worth exploring as an approach despite wider evidence pointing to targeted interventions [68].

Similarly, the identification and selection of students as part of a targeted approach can create stigma, and the potential barrier to help-seeking and participation as a result [12]. Universal interventions may avoid this by taking a full-student population approach. There is potential in universal approaches for academic institutions to integrate everyday activities such as walking, requiring minimal equipment, facilities and added cost. Designing campuses and curriculums with increased opportunity and encouragement of PA for full-student populations may be particularly important as there has been concern around the prevalence of poor mental health in subgroups of adolescents and young adults, such as females [116, 117]. Gender differences in preferences and attitudes towards PA are important factors in engagement with and benefits of PA [118]. Just two studies reported an analysis of the effects between genders, both of which reported yoga-based interventions had more prominent effects for females compared to males; while one reported that male participants rated yoga less beneficial and school sports more beneficial, the opposite was true for their female colleagues [73, 99]. The lack of research on gender differences (and indeed other inequalities) identified in this review further emphasises the need for routine measurement and assessment of these factors to inform future intervention development to ensure the minimisation of intervention generated inequalities [119]. This information would strengthen decisions around whether a universal or targeted approach to PA interventions for mental health are most appropriate in educational settings.

### Practical implications for interventions within the education setting

The findings suggest that upper secondary/tertiary education settings are acceptable and feasible options for the delivery of PA-based interventions to support the mental health of young people. Many schools and universities have facilities and trained sport and exercise staff available. Strategies to increase compliance and attendance unique to the education setting can also be implemented, such as making attendance mandatory as part of the curriculum or providing external rewards in the form of academic credits. Similarly, delivering PA-based mental health interventions within the education setting may help overcome typical barriers blocking access to treatment. For example, provided these types of interventions do not result in financial cost to the student or their family, they could increase accessibility for those who may be from lower socioeconomic backgrounds [12]. While the education setting may be a more familiar and acceptable context to encourage help-seeking and reduce stigma amongst students compared to traditional clinical settings, it is important to note that socioeconomic barriers will still apply to PA [12]. Further student-related barriers may affect the implementation of PA-based interventions within education settings, such as low confidence to engage in physical activity, and negative attitudes towards PA participation within the academic setting [6]. As reported as a significant positive characteristic of two interventions included in this review, incorporating choice and catering for different participant’s preferences and perceived skill levels for a variety of PA types are important when designing PA-based interventions to minimise the potential for stigma in relation to PA participation [120].

### Strengths and limitations

This review is the first to examine the range of evidence available regarding physical activity-based interventions to improve mental health-related outcomes in young people aged 16 to 25 within education settings. This review was conducted and reported in accordance with scoping review guidance (PRISMA) and the review protocol was pre-registered. However, there are some limitations. As this review included studies conducted between 2015 and 2025, there may have been relevant studies conducted before this timeframe that could have contributed to the findings. This review included studies conducted in high income countries only, so there may have been relevant literature from low- or middle-income countries that have been missed. Similarly, keywords related to inequalities, such as physical or cognitive disabilities or socioeconomic background were not included, so it may be that papers might have been missed without the use of these keywords. While a meta-analysis was outside the scope of this review, the mixed quality of evidence and heterogeneity in both intervention design and outcome measurement limited the extent to which comparisons could be made between studies and evidence of effectiveness of interventions pooled. As presented in Table 3, several further recommendations for future research have been identified.

### Conclusions

The findings of this scoping review suggest PA is a potentially effective intervention option to support the mental health of young people in upper secondary and post-secondary education settings. A wide variety of types of PA and intervention characteristics were identified. Beneficial effects of PA interventions were reported for a range of mental health-related outcomes, particularly depressive symptoms, anxiety and stress. A lack of research was identified assessing the impact of interventions on population subgroups, including research with older adolescents, research comparing outcomes by gender or socioeconomic background and research investigating the effects of interventions on young people with learning or physical disabilities. The results of this review, such as the characteristics of interventions and barriers and facilitators identified, are useful to inform the development of future PA interventions within education settings. PA may be an effective option as both a universal mental health promotion strategy and as a mental health treatment option integrated within the mental health services of academic institutions.

**Table 2:** Overview of the characteristics of the included studies, the physical activity-based interventions they examined and a summary of results reported

Characteristics of included studies							
Study	Study design	Education setting and location	Participants (sample size, age, sex); experimental group distribution (*n*)	Universal/ targeted	PA intervention overview	Outcomes assessed (outcome measure)	Summary of findings
Arbinaga et al., 2018 [53]	RCT	Post-secondary education; University. Huelva, Spain.	**Total N** = 114 **M age** = 19.81 (SD = 1.75) **Sex**: M = 14.9%, F = 85.1%. **Groups** : **Non-intervention control**: *n* = 13. **Int 1**: *n* = 15. **Int 2** : *n* = 17. **Int 3**: *n* = 21.	Universal	**Int 1**: moderate-intensity aerobic exercise intervention **Int 2**: moderate-intensity aerobic exercise + negative manipulation of expectations: there would be no psychological benefits of the intervention **Int 3**: moderate-intensity aerobic exercise + positive manipulation of expectations: there would be psychological benefits of the intervention All provided 3 × 70-min group sessions per week for 7 weeks, supervised by a fitness instructor at the University sports facilities.	Self Esteem (RSE)	Self-esteem was improved in all 3 intervention groups compared to control. Medium effect sizes for Int 1 and Int 2, and large effect size for Int 3 were presented; however specific effect sizes were unable to be extracted.
						Wellbeing (Positive well-being subscale from Subjective Exercise Experiences Scale (SEES))	There was a within group increase in wellbeing reported for intervention group 3 only, with a moderate to large effect size reported. Effect size unable to be extracted.
Balciunene et al., 2022. [66]	Quasi-exp	Post-secondary education; University. Kaunas, Lithuania.	**Total N** = 110 **M age** = 21.5 (Sd = 3.5). **Sex**: F = 100%. **Groups**: **Non-intervention control**: *n* = 80. **Int group**: *n* = 30.	Universal	‘Body and Mind’ CBT and mindfulness-based PA intervention of mixed-PA types including Pilates, yoga and resistance training. Reported mixed intensities from low to high in resistance sessions. 1 × 60-min group session per week for 8 weeks, supervised by fitness instructor at a local fitness centre.	Body dissatisfaction (EDI 2)	Body dissatisfaction was decreased (*p* = 0.003) after the intervention compared to control. Cohen’s d= −0.4.
						Body image (positive) (BAS-2).	BAS-2 scores increased (*p* = 0.001) in the intervention group compared to the control, d = 0.7.
						Body image (concerns): (SATAQ-4 and MBSRQ-AS).	SATAQ-4 scores were reduced (*p* = 0.004) after the intervention compared to control, d = 0.6. There was a reduction(*p* < 0.05) in the appearance orientation (d = 0.9), and an increase in the appearance evaluation (d = 0.5) and body satisfaction (d = 0.7) subscales of the MBSRQ-AS compared to control.
						Stress (Reeder Stress Inventory)	Stress marginally decreased for both the intervention and control groups, with no difference between groups. No effect size reported.
Brandão et al., 2024 [72]	RCT	Post-secondary education; University. Vila Real, Portugal.	**Total N =** 106. **M Age**: 21.09 **Sex**: M = 18.86%, F = 81.14%. **Passive control group**: *n* = 44 **Active control: “autogenic training”**: n = 34. **Yoga Intervention group**: *n* = 28	Universal.	Yoga intervention based on Kundalini yoga. 1 × 60-min group yoga session for 6 weeks. Sessions were delivered virtually via Zoom, and delivered by an external yoga instructor certified in this yoga form.	Depressive symptoms, Anxiety and Stress (all measured using the DASS-21); Emotion regulation (Emotion Regulation of Others and Self); Self-compassion (Self-compassion Scale); Self-concept (self-concept clinical inventory); Subjective happiness (Subjective Happiness Scale)	Depressive symptoms, anxiety and stress were significantly reduced at post-intervention for all 3 groups, however, there were no significant differences between groups. There were significant improvements in participant’s self-compassion and emotion regulation in the yoga intervention group compared to the two control groups.
Castellote-Caballero et al., 2024 [84]	RCT.	Post-secondary education; University. Las Palmas de Gran Canaria, Spain.	**Total N =** 129. **M age**: 20.29. **Sex**: M = 48.8%, F = 51.2%. **Intervention group**: *n* = 65. **Control group**: *n* = 64.	Universal.	Yoga intervention comprised of 2 × 60-min yoga sessions per week for 12 weeks. Sessions were delivered by a physiotherapist certified in yoga instruction.	Anxiety (STAI); Stress (PSS); Wellbeing (WEMWBS);	The yoga intervention had significant positive effects on all outcomes compared to the control group between pre and post intervention, with small effect sizes for wellbeing (Cohen’s d = 0.47), state anxiety (Cohen’s d = 0.38) and stress (Cohen’s d = 0.44) and a medium effect size for trait anxiety (Cohen’s d = 0.80).
Cecchini-Estrada et al., 2015. [57]	RCT	Post-secondary education; University. Oviedo, Spain.	**Total N =** 106 **M age**: 19.61 (SD = n/a). **Sex**: M = 36%, F = 64%. **Groups**: **Active**,** attention control group (stretching)**: *n* = 26. **Int 1**: *n* = 26. **Int 2**: *n* = 27. **Int 3**: *n* = 27.	**Targeted**: participants recruited for being sedentary and experiencing elevated depressive symptoms.	**Int 1**: Aerobic exercise intervention (unsupervised) **Int 2**: Aerobic exercise intervention (supervised: PE instructor) **Int 3**: Aerobic exercise intervention supervised & based on TARGET principles (Task, Authority, Reward, Groupings, Assessment, Time) All moderate-to-vigorous intensity. 3 × 60-min sessions, for 8 weeks.	Depressive symptoms (Adapted Depressive Mood Scale).	Depressive symptoms improved for all groups between pre and post intervention, and depressive symptoms were reduced in all 3 intervention groups compared to control between pre and 6-month follow up, with Int 3 reporting the most prominent reduction effects. Hedges g = 0.7 (Int 1), 0.77 (int 2), 1.7 (Int 3).
Chauhan et al., 2024 [75]	Pilot study.	Post-secondary education; University. Pécs, Hungary.	**Total N =** 28. **M age**: 23.54 (sd: 4.36) **Sex**: M = 21.4%, F = 78.6%. **Intervention group**: *n* = 28. No control group or comparator	Universal.	“GSY Goodbye Stress with Yoga”: Yoga intervention consisting of 1 × 90-min yoga session per week for 10 weeks. The sessions were delivered by a certified yoga instructor in the university’s gym hall.	Anxiety, Depression and Stress (All DASS-21); Quality of life (WHOQOL-Bref)	DASS-21 scores were significantly decreased after the intervention for anxiety (*p* = 0.019) and depressive symptoms (*p* = 0.049), and while stress scores were decreased, this was not a statistically significant change (*p* = 0.078). Effect sizes not reported. Physical health domain and psychological health domain scores of the WHOQOL-Bref were significantly improved after the intervention (*p* = 0.001 and p = < 0.001). The other domains, social relation, environmental and quality of life, were improved but these changes were not statistically significant.
Ciezar Andersen et al., 2024. [51]	Quasi-exp.	Post-secondary education; Universities (*n* = 6). Alberta, Canada.	**Total N =** 68. **M age**: not stated (74% aged 17–25, 26% aged 26 and older) **Sex**: M = 6%, F = 94%. **Intervention group**: *n* = 68. No control group or comparator.	Universal.	Yoga intervention in which participants were provided with pre-recorded, video yoga classes of low to moderate intensity and encouraged to complete 3 classes per week in their own time for the six week intervention period. Yoga classes ranged from 25 to 67 min (m = 45 min) and were designed and recorded by a certified yoga practitioner with 20 years’ experience (who was also the lead author).	Anxiety, depressive symptoms and Stress (all DASS-42); Self-compassion (self-compassion scale);	There was significant improvements in DASS-42 scores after the intervention, with large effect sizes for anxiety (*p* < 0.001, Cohen’s d = 1.11), depressive symptoms (*p* < 0.001, cohen’s d = 1.04) and stress (*p* < 0.001, cohen’s d = 1.34). Self-compassion was significantly increased after the intervention, with a large effect size (*p* < 0.001, cohen’s d = 1.15).
Cioffi & Lubetzky, 2023 [86]	Pilot study.	Upper secondary education; public high school. New York, U.S.A.	**Total N =** 42. **M age**: 15.7. **Sex**: M = 31%, F = 69%. **Int group: “BOXVR”** n = 14. **Comparator group: guided video exercise** *n* = 14. **Control group**: *n* = 14.	Universal.	**Int 1: “BOXVR”**: Participants were provided with an “Oculus Rift” virtual reality headset and earpiece, loaded with the game BOXVR which involves participants having to duck/squat to avoid incoming virtual obstacles and punch virtual targets. Participants engaged of 5 × 10-min BOXVR sessions per week for 3 weeks. Sessions took place in a quiet classroom at the school during school hours. **Comparator Int**: Guided video boxing exercise: Participants were provided with a pre-recorded guided video exercise programme that consisted of aerobic movements similar to that experienced while playing BOXVR (squats, ducks, punches), and engaged in 5 × 10 min guided video sessions per week for 3 weeks. Sessions took place in a quiet classroom at the school during school hours. **Control group**: received no intervention.	Anxiety (Pediatric Anxiety Short Form); Stress (Psychological Stress Experience Short Form 8a).	The BOXVR group showed a significant reduction in stress scores after the intervention compared to the guided video exercise and control groups; effect size not reported. There was no difference between groups on anxiety scores after the intervention.
Cox et al., 2017. [99]	Pilot study	Upper secondary education; High school. Northwest USA.	**Total N =** 47. **M age**: 16.45 (sd = 1.0). **Sex**: M = 10%, F = 90%. **Groups**: **PE as usual control**: *n* = 23. **Int group**: *n* = 20.	Universal	Yoga programme to supplement PE classes. Intensity not specified. 1 × 40-min & 1 × 75-min group yoga session in addition to 2 x PE sessions per week for 12 weeks. Sessions were supervised by a PE teacher and yoga instructor and delivered within the education setting.	Body image (BAS-2).	There was a minimal increase in body appreciation after the yoga intervention and PE as usual control, with no differences in effects between groups (*p* = 0.418, η²ρ = 0.02).
						Body surveillance (Objectified Body Consciousness Scale)	Body surveillance was moderately decreased after the yoga intervention but not in the PE as usual control. (*p* < 0.05, η²ρ = 0.19).
						Self-worth (PSDQ)	There was a minimal increase in self-worth in the full sample compared to the control group (*p* = 0.108, ηp²= 0.06). There was a minimal-to-moderate increase in self-worth in a sub-analysis conducted on females only, (*p* = 0.44, ηp²= 0.13).
Daly et al., 2015. [100]	RCT	Upper secondary education; High school. New York, USA.	**Total N =** 37. **M age =** 16 (sd n/a). **Sex**: M = 62.2%, F = 37.8%. **PE as usual control**: *n* = 18. **Int group**: *n* = 19.	Universal.	“Bent on Learning” Yoga intervention. Intensity not specified. 3 × 40-min group yoga sessions per week for 16 weeks, delivered within the education setting by an external yoga instructor.	Emotion regulation (ERICA)	Emotion regulation increased after the yoga intervention but not for PE as usual control. No effect size reported.
Davis et al., 2024. [76]	Pilot study.	Upper secondary education; High school (*N* = 2; 1 x rural town high school and 1 x school in very remote, rural location). Montana, U.S.A.	**Total N =** 40. **M age**: not stated (age range 15 to 17). **Sex**: M = 50%, F = 50%. **Int group 1**: ‘Face-to-face’ rural town high school group: *n* = 18. **Int group 2**: Remotely delivered intervention group for very rural school: *n* = 22.	Universal.	Trauma-informed yoga intervention consisting of 2 × 45-min yoga sessions per week for 6 weeks delivered instead of student’s regular PE class. For the face to face intervention group, sessions were delivered in-person by a certified yoga instructor, while the remote group received the sessions virtually led by another certified yoga instructor.	Anxiety (GAD-7)); Depressive symptoms (PHQ-A); Resilience (Connor-Davidson Resilience Scale).	While there were non-significant improvements in anxiety for both intervention groups, and in resilience for the face to face intervention group, depressive symptoms was the only outcome with statistically significant improvements. PHQ-A depressive symptom scores were significantly reduced after the intervention for the face to face intervention group (*p* < 0.01) and for the remotely delivered intervention group (*p* < 0.05).
de Vries et al., 2016 & deVries et al., 2018. [47, 48]	RCT	Post-secondary Education; University. Nijmegen, The Netherlands.	**Total N =** 99. **M age** : 20.86 (sd = 2.3). **Sex**: M = 19.2%, F = 80.8%. **Wait list control group**: *n* = 49. **Int group**: *n* = 50	**Targeted**: recruited students who exceeded cut off for high levels of fatigue.	Low intensity aerobic exercise intervention – outdoor running. 3 × 60-min group running sessions per week for 6 weeks, supervised by a licenced running therapist.	Emotional exhaustion/burnout (Utrecht Burnout Scale)	There were no differences in effects on emotional exhaustion after the exercise intervention compared to control.
						Self-efficacy (General Self Efficacy Scale (GSES-12) and self-report visual analogue scale measure of self-efficacy)	There was no effect on self-efficacy as measured with the GSES-12 reported after the intervention (*p* > 0.05). η²= 0.02. Self-efficacy as measured using the single item VAS increased for participants with higher exposure/adherence to the exercise intervention while it did not for those with lower exposure/adherence.
DeJong et al., 2021. [6]	Mixed methods.	Post-secondary education; University. Canada.	**Total N =** 91. **M age**: 22.96 (sd = 2.3). **Sex**: M = 18%, F = 82%. (No control group)	**Targeted**: students referred from campus mental health services for mild to moderate mental health concerns	Individualized PA behaviour change coaching and PA training intervention of mixed aerobic and resistance exercise supervised by a PA trainer. Intensity was self-selected. 1 × 60-min individual session per week for 6 weeks.	Anxiety (MHI-38)	Anxiety was reduced in university students referred from campus mental health services (*p* < 0.001), with a medium effect size, Cohen’s d = 0.75.
						Depressive symptoms (MHI-38)	Depressive symptoms were reduced after the intervention. Cohen’s d = 0.67. (1.11; −0.52).
						Psychological distress (MHI-38).	Psychological distress was decreased (*p* < 0.001) after the intervention. Cohen’s d = 0.78 (−0.88, −0.46).
Eather et al., 2019. [62]	RCT	Post-secondary education; University. Newcastle, Australia.	**Total N =** 53. **M age**: 20.38 (sd = 1.88). **Sex**: M = 34%, F = 66%. **Wait list control group**: *n* = 31. **Int group**: *n* = 22.	Universal.	‘Uni-HIIT’ high intensity interval training intervention. High intensity - >85% of MaxHR. Sessions increased from 8 to 10 to 12 min as intervention progressed, 3 group sessions per week for 8 weeks delivered by a researcher within the education setting.	Anxiety (STAI)	There were no differences in effects on anxiety in university students after the intervention compared to control. Cohen’s d = 0.02.
						Stress (Perceived stress scale)	There was a small beneficial effect of the intervention on anxiety, although there were no differences in effects between the intervention and control (*p* > 0.05, Cohen’s d = 0.20).
Elstad et al., 2020. [92]	RCT.	Post-secondary education, university. Oslo, Norway.	**Total N =** 202. **M Age** = 25.32 (sd = n/a). **Sex**: M = 13%, F = 87%. **Control**: *n* = 102 **Int group**: *n* = 100	Universal.	Yoga-based intervention. Intensity not specified. 2 × 75-min group yoga sessions per week for 12 weeks supervised by a yoga instructor and conducted in a local yoga studio.	Life satisfaction (SWLS)	Life satisfaction increased after the yoga intervention compared to control between pre and post-intervention (Cohen’s d = 0.36), which was maintained until 6-month follow up (Cohen’s d = 0.22).
						Psychological distress (HCL-25).	Psychological distress was decreased at both post intervention and six month follow up compared to control. Post: Cohen’s d= −0.32. 41% decrease (risk ratio 0.59, 95% CI: 0.35 to 0.95) Follow up: Cohen’s d= −0.38. 33% decrease in risk of scoring above 1.75 cut off value (risk ratio 0.67, 95% CI 0.39 to 1.03)
						Wellbeing (WEMWBS)	Wellbeing was moderately increased as measured by the WEMWBS compared to control at post intervention. Cohen’s d = 0.36. While the increase was maintained at follow up, it was no longer different to control. Cohen’s d = 0.07.
Falsafi, 2016. [70]	RCT.	Post-secondary education; University. South-eastern USA.	**Total N** = 90. **M age**: 22.1 (sd = n/a). **Sex**: M = 13.6%, F = 86.4%. **Control group**: *n* = 30 **Non-PA Comparator (mindfulness intervention)**: *n* = 30. **Int group**: *n* = 30.	**Targeted**: recruited students with a diagnosis of depression or anxiety.	Yoga-based intervention. Intensity not specified. 1 × 75-min group yoga session per week for 8 weeks, delivered by a certified yoga instructor in the University recreation centre.	Anxiety (Hamilton Anxiety Scale)	There were significant reductions in Anxiety (*p* < 0.01) in university students with a diagnosis of depression or anxiety at post (week 8) and follow-up (week 12) after both the yoga and the non-PA comparator mindfulness intervention compared to control. No effect size reported.
						Depressive symptoms (BDI)	There were significant reductions in Depressive symptoms(*p* < 0.01) in university students with a diagnosis of depression or anxiety at post (week 8) and follow-up (week 12) after both yoga and comparator mindfulness interventions compared to control. Effect size not reported.
						Self-compassion (Self-Compassion Scale)	There was no effect on self-compassion after the yoga intervention. However, there was an increase in self-compassion after the non-PA comparator mindfulness intervention to post-intervention, which was maintained until follow up. No effect size reported.
						Stress (Student-Life stress inventory)	Stress was reduced in university students with a diagnosis of depression or anxiety at post (week 8) and follow-up (week 12) after both the yoga and non-PA comparator mindfulness interventions compared to control. No effect size reported.
Forseth et al., 2022. [81]	Feasibility study	Post-secondary education; University. Milwaukee, USA.	**Total N =** 14. **M age**: 23.8 (sd = 4.6). **Sex**: M = 29%, F = 71%. (No control).	Universal.	Yoga-based intervention. Intensity not specified. 2 × 60-min group yoga sessions per week for 8 weeks. Delivered within the education setting and supervised by an external yoga instructor.	Depressive symptoms (BDI) .	Depressive symptoms changed between the time points (pre: 10.3 ± 8.4, to mid-intervention: 5.7 ± 6.3, to post: 7.7 ± 8.0). Cohen’s d = 0.36, 21% power for change in BDI-II. Sub-analyses also reported larger reductions in depressive symptoms among participants who did not meet guideline amounts of PA at baseline compared to those who did.
						Stress (PSS)	Changes in perceived stress were reported; pre: 16.2 ± 8.0 to mid: 12.8 ± 7.4 to post: 14.3 ± 8.5. Cohen’s d = 0.49 with 48% power for change in PSS. Sub-analyses also reported larger reductions in stress among participants who did not meet guideline amounts of PA at baseline compared to those who did.
Friedman et al., 2022; Marenus et al., 2021; Murray et al., 2022. [58, 60]	Quasi-exp	Post-secondary education; University. Michigan, USA.	**Total N =** 77. **M age**: 23.43 (sd = 6.0). **Sex**: M = 5%, F = 84%, Transgender & non-conforming (TGNC) = 7%. (No Control group) **Int 1 WeActive**: *n* = 43. **Int 2 WeMindful**: *n* = 28.	Universal.	**Int 1: ‘WeActive’** Moderate-to-vigorous intensity aerobic and resistance exercise intervention. Delivered in groups virtually via ZOOM, supervised by a strength and conditioning specialist. **Int 2: ‘WeMindful’** mindfulness-based yoga intervention. Intensity not specified but likely low intensity. Delivered in groups virtually via ZOOM, supervised by a movement science student. Both provided 2 × 30-min sessions per week for 8 weeks.	Anxiety (Generalized Anxiety Disorder Scale)	Both groups reported an improvement in anxiety scores after the intervention, however these were not statistically significant.
						Depressive symptoms (Major Depression Inventory)	Both WeMindful and WeActive groups reported a significant decrease in Depressive symptoms after the interventions.
						Resilience (CD-RISC-10)	Resilience was increased in both intervention groups at post intervention and was maintained but did not further increase between post intervention and 6-week follow up. No effect size reported.
						Subjective vitality (Subjective Vitality Scale)	There was an increase in Subjective vitality after both the WeMindful and WeActive intervention groups (η²= 0.088). The WeMindful intervention had more prominent increases in subjective vitality.
						Wellbeing (WHO-5)	There was an increase in Wellbeing after the WeMindful intervention, however, wellbeing slightly decreased after the WeActive intervention.
Fukui et al., 2021. [94]	RCT.	Post-secondary education; University. Hiroshima, Japan.	**Total N =** 125 **M Age**: 21.6 (sd = 2.9) **Sex**: male = 45.6%, female = 54.4%. **Intervention group**: *n* = 60. **Control group**: *n* = 60.	Universal.	A self-guided “stay-at-home” exercise video intervention provided to participants in lockdown during the COVID-19 pandemic. Participants were provided a link to a platform which hosted videos and instructed to watch and follow along to a different 5-min guided exercise video every day for 8 weeks. Videos were 5-mins in duration and each video targeted a different body group, either upper limb, lower limb or core stability exercises. There were 7 videos pre-recorded for each body group; videos were designed and recorded by a physical therapist.	Quality of Life (SF-8); Wellbeing (WHO-5); Psychological distress (Kessler Screening Scale for Psychological Distress (K6)).	There was a significant improvement after the intervention for Wellbeing (*p* < 0.05) and psychological distress (*p* < 0.01) compared to the control group. Effect sizes not reported. While no significant effects were reported for any of the physical function, role physical, bodily pain, vitality, social function, or mental health subscales of the SF-8, there was a significant improvement in the general health subscale (*p* < 0.01) in the intervention group compared to control. No effect size reported.
Glaser et al., 2022. [96]	Quasi-exp	Upper secondary education; vocational schools. Israel.	**Total N =** 56. **M Age =** 16.18 (sd = 0.83). **Sex**: M = 91%, F = 9%. **Waitlist control**: *n* = 29. **Int group**: *n* = 27.	**Targeted**: recruited ‘at-risk’ youth in vocational secondary schools.	Online Mentoring Health Intervention, an individualized intervention combined virtual health coaching and physical activity, supervised by multidisciplinary team. 1 x one-to-one session comprised of 30-mins PA and 30-mins tutor coaching per week for 8 weeks delivered virtually.	Perceived social support (MSPSS)	Perceived social support increased after the intervention compared to control (Cohen’s d = 1.11 (95% CI 0.52 to 1.97).
						Psychological distress (Brief Symptom Checklist (BSI))	Psychological distress was decreased after the individually tailored intervention compared to control. Cohen’s d= −1.03 (95% CI −1.67 to −0.42).
						Resilience (Brief resilience scale (BRS))	Resilience increased after the intervention compared to control. Cohen’s d = 1.88 (95% CI 1.07 to 2.81).
Gurung et al., 2023. [77]	Pilot study.	Post-secondary education; University. Sheffield, UK.	**Total N =** 24. **M age**: not reported. **Sex: m** = 21%, **f** = 79%. **Intervention group**: *n* = 24. **No control group.**	Hybrid Universal and Targeted; advertisements were circulated across all-university students, while participants were also referred from campus mental./physical health services for those seeking help for with mental health issues.	“Mindfit”: a 10-week couch to 5k therapeutic running group programme. There was one weekly running session of 60-mins conduced outside in nature in a group setting, and this was combined with psychoeducation and counselling which took place directly after the running session each week, for 55 min and included socialising with drinks/snacks. The intervention was directed by the university wellbeing service and facilitated by a team of counsellors, mental health nurse, psychologists and a sports manager.	Anxiety (GAD-7); Depressive symptoms (PHQ-9). Qualitative data: focus groups were conducted with participants to explore the impacts of the intervention.	There were significant improvements in anxiety (*p* < 0.05) and depressive symptoms (*p* < 0.01) after the intervention. No effect size reported, however, scores on both scales were decreased by almost 50%. Qualitative data reported positive impacts of the intervention in three themes; creating a safe community, creating a pathway to success and making progress. Participants noted the benefits of the sense of community and reductions it had on their individual anxiety. They reported the benefits of the safe space and relating to others and how it improved their sense of belonging to the university. They also reported improvements in their self-belief and mastery with the progress they felt they were making during the intervention.
Herbert et al., 2020. [52]	Pilot study.	Post-secondary education; University. Germany.	**Total N =** 91. **M age**: 22.84 (sd = 3.54). **Sex**: M = 10%, F = 90%. **Waitlist control**: *n* = 31. **Non-PA Comparator (expressive writing intervention)**: *n* = 24. **Int group**: *n* = 19.	Universal.	Low-to-moderate intensity aerobic exercise intervention consisting of bodyweight aerobic movements, including running on the spot, arm punches and high knees. Sessions increased from 8-mins to 12-mins during the intervention, 2 sessions per week for 6 weeks. Sessions were unsupervised and students completed them independently, to a follow along pre-recorded exercise video made bespoke for the study.	Anxiety (STAI)	There were no significant effects on anxiety between pre and post intervention in any of the groups, although the exercise intervention group’s state anxiety subscale scores were lower than the non-PA comparator expressive writing intervention and control groups at post-intervention.
						Body dissatisfaction (EDI-2).	There was a small decrease in body dissatisfaction for all participants between pre and post intervention, no differences in effects between groups (η²= 0.004).
						Depressive symptoms (BDI).	Depressive symptoms were reduced (*p* < 0.005) after the aerobic exercise intervention, Cohen’s d = 0.62, while scores increased in the non-PA comparator group and there were no changes in the control group. In the aerobic exercise intervention group, 73.86% demonstrated improvements in depressive symptoms between pre and post intervention.
						Positive and negative affect (PANAS)	Positive affect decreased and negative affect increased in the intervention, non-PA comparator and control groups between pre and post intervention.
						Stress (SCI)	There was a reduction in overall perceived stress (full SCI sum score) between pre and post for all participants, however this was most pronounced in the exercise intervention group, Cohen’s d = 0.72. Stress due to uncertainty subscale scores were decreased in the aerobic exercise intervention group but not in the control or non-PA comparator, Cohen’s d = 0.77.
						Wellbeing (WHOQOL-BREF)	There were no differences in effects on wellbeing between groups between pre and post intervention.
Hsu et al., 2021. [78]	Quasi-exp	Upper secondary education; High school. Taiwan.	**Total N =** 64. **M age**: 16.7 (sd = 0.8). **Sex**: M = 37.5%, F = 62.5%. (No control group)	Universal.	Daily brisk walking intervention targeting 30-mins per day with an education component at the beginning of the intervention. Brisk walking intervention lasted for 12 weeks.	Anxiety (Beck youth Inventory-2)	Anxiety was decreased between pre and post brisk walking intervention, with onset of the decrease observed from week 6 of the 12-week intervention. There was a positive correlation between amount of brisk walking and decrease in anxiety (*r* = 0.36), with the authors reporting brisk walking of 60 min per week was more effective in decreasing anxiety than shorter amounts.
						Depressive symptoms (Beck youth Inventory-2)	Depressive symptoms were decreased between pre and post brisk walking intervention, with onset of the decrease observed from week 6 of the 12-week intervention. There was a positive correlation between amount of brisk walking and decrease in depressive symptoms (*r* = 0.31), with the authors reporting brisk walking of 60 min per week was more effective in decreasing anxiety than shorter amounts.
						Self-concept (Beck youth Inventory-2)	Self-concept was increased between pre and post brisk walking intervention, with onset of the increase observed from week 12 of the 12-week intervention. However, there was no correlation between the amount of brisk walking and self-concept.
Jeftic et al., 2023. [82]	Feasibility study.	Post-secondary education; University. Perth, Australia.	**Total N =** 114. **M age**: 23.7 (sd = n/a). **Sex**: M = 31.1%, F = 66.6%, non-binary = 1.8%. (No control group)	**Targeted**: participants referred from a campus mental health service for a diagnosis of a mental health condition.	STRIDE programme: Individualized exercise intervention delivered supervised by a mentor; participants engaged in individually tailored exercise including PA types resistance training, aerobic exercise, sports, mobility and fitness classes. Individualized: minimum of 1 session per week for 12 weeks.	Depressive symptoms (QIDS-SR-16)	A mean reduction of 3.87-points in QIDS-SR-16 was reported between pre and post intervention, a clinically meaningful reduction in depressive symptoms (above the threshold of 3.75 for this scale). Cohen’s d= −0.78.
						Quality of life (Short Form-12 (SF-12))	There was no effect on the physical health subscale of the quality-of-life measure (d = 0.06), however, scores on the mental health subscale increased after the intervention, with a 38.76% pre-to-post increase, Cohen’s d = 1.04.
						Self-efficacy (Response, Lifestyle, Barrier and Exercise Self Efficacy subscales).	There were negligible effects (d = 0.08) on response efficacy (a student’s confidence exercise would result in mental health improvement). There was a small-to-medium increase (Cohen’s d = 0.39) in lifestyle self-efficacy (a student’s confidence in their ability to change lifestyle behaviours impacting on their mental health), and a medium increase (Cohen’s d = 0.53) in barrier self-efficacy (a student’s confidence in their ability to overcome exercise barriers). There was a large increase in exercise self-efficacy (a student’s confidence in their capability to engage in exercise), Cohen’s d = 1.19.
Jeitler et al., 2020(a); Jeitler et al., 2020 (b). [73, 101]	Pilot study	Upper secondary education; vocational secondary school. Germany.	**Total N =** 102. **M age**: 19.6 (sd = 2.2). **Sex**: M = 47%, F = 53%. **PE as usual control**: *n* = 38. **Int group**: *n* = 54.	Universal.	Yoga-based intervention. 1 × 90-min group yoga session per week for 10 weeks, delivered within the education setting and supervised by an external yoga instructor.	Anxiety (HADS)	Anxiety was reduced after the yoga intervention between baseline and post intervention, however, the anxiety scores almost returned to baseline by 6-month follow up.
						Depressive symptoms (HADS)	There was a minimal, nonsignificant reduction in depressive symptoms in the yoga intervention group between baseline and post intervention, however, scores returned to higher than baseline at 6-month follow up.
						Stress (PSS)	Stress was reduced after the yoga intervention between baseline and post intervention, although stress scores almost returned to baseline at 6 month follow up. Students also reported beneficial stress reduction effects of the intervention in focus groups.
						Wellbeing (WHO-5)	There were no effects on wellbeing in either the yoga intervention or PE as usual groups. However, students qualitatively reported perceived beneficial effects on psychological wellbeing in focus groups.
Kahlin et al., 2016. [98]	cRCT.	Upper secondary education; high school. Sweden.	**Total N =** 104. **M age**: 17.2 (sd = 0.55). **Sex**: F = 100%. **PE as usual control**: *n* = 44. **Int group**: *n* = 60.	**Targeted**: recruited sedentary/physically inactive female students.	Individualized intervention; participants provided with membership to local fitness centre and provided guidance and encouragement to engage in at least 1 PA session (type and intensity self-selected) per week for 24 weeks.	Self-esteem (CY-PSPP)	There were significant beneficial effects on self-esteem after the PA intervention between pre and post intervention, which were maintained at 1-year follow up.
						Self-worth (CY-PSPP)	Self-worth was increased in the PA intervention and control group between pre and post intervention, although there were more prominent effects in the intervention group.
Kim., 2024 [80].	Quasi-exp.	Post-secondary education; University. Gwangju, Republic of Korea.	**Total N =** 102. **M age =** 20.45. **Sex**: Males = 1.96%; Females = 98.03%. **Intervention group**: *n* = 50. **Control group**: *n* = 52.	Universal.	‘WalkON’: a mobile-device health management application used to promote walking. For 12 weeks, participants used this program to build healthy habits and were instructed to walk for at least 30 min per day for a minimum of 5 days per week for the 12-week period. Engagement was also supported through step-count monitoring, access to health education materials on the WalkOn platform, and incentivizing step challenges.	Anxiety (BAI); Depressive symptoms (PHQ-9); Self-efficacy (Self-efficacy scale); Social support (Lubben Social network scale).	The intervention group experienced a significantly bigger decrease in anxiety scores (*p* = 0.20; effect size not reported) and increase in self-efficacy (*p* = 0.046; effect size not reported) compared to the control group Depression scores were decreased in both groups, however, these were not statistically significant changes. There was no significant effects on social support for either group.
Latino et al., 2021. [61]	RCT	Upper secondary education; High school. Bari, Italy.	**Total N =** 60. **M Age**: 16.13 (sd = 0.74). **Sex**: F = 100%. **PE as usual control**: *n* = 30. **Int group**: *n* = 30.	**Targeted**: recruited female adolescents with obesity defined as a high BMI.	Supervised exercise programme consisting of moderate-to-vigorous intensity bodyweight aerobic exercise movements (40 min) along with flexibility (10 min) and competitive games (30 min). 3 × 90-min group sessions per week for 12 weeks, conducted at the end of the school day.	Self-efficacy (GSES-12)	There was a significant improvement in self-efficacy in the intervention group, with a large effect size, d = 2.34. There was no changes in self-efficacy in the control group.
Lavadera et al., 2020. [54]	Quasi-exp.	Post-secondary education; University. New Jersey, USA.	**Total N =** 61. **M age**: 24 (sd = n/a). **Sex**: M = 37%, F = 63%. **Non-intervention control**: *n* = 30. **Int group**: *n* = 17.	Universal.	‘MAP: Train my brain’ meditation and moderate intensity aerobic exercise intervention. 2 × 30-min group sessions per week for 8 weeks, supervised by certified aerobic training instructor and senior researcher and delivered within the education setting.	Depressive symptoms (PHQ-8)	There was a minimal decrease in depressive symptoms after the intervention (*p* > 0.05).
						Quality of life (QOLS)	Quality of life was significantly increased by 7% after the PA intervention, while it was slightly decreased in the control group.
						Rumination (Ruminative Response Scale (RRS))	Overall ruminative thoughts were reduced by 17% and depressive rumination and brooding rumination subscales were reduced by 16% and 24% respectively after the PA-intervention, with no changes in the control.
						Stress (PSS)	The PA intervention had significant beneficial effects on stress, while there were no changes in stress in the control group.
Lemay et al., 2019. [85]	Pilot study.	Post-secondary education; University. Rhode Island, USA.	**Total N =** 20. **M age**: 20.7 (sd = 1.2). **Sex**: M = 23.5%, F = 76.5%. (No control)	Universal.	Yoga and meditation intervention. 1 × 90-min group session per week for 6 weeks. Delivered within the education setting and supervised by a researcher.	Anxiety (BAI)	Anxiety was decreased by a mean 9.6 points (95% CI: −13.8, −5.3). Categorical analysis found no participants were categorised as high anxiety at post intervention (*p* = 0.008) where 29.4% were at baseline.
						Stress (PSS)	Perceived stress decreased 7.9 points (95% CI: −11.8, −4.0) after the yoga intervention. Categorical analysis of PSS found no students were categorised as high stress at post intervention (*p* = 0.03), where 29.4% were at baseline.
(López-Rodríguez et al., 2017) [71]	RCT.	Post-secondary education; University. Almeria, Spain.	**Total N =** 121. **M age =** 22.33(sd = 4.12). **Sex**: M = 25.3%, F = 74.7%. **Waitlist control**: *n* = 61. **Int group**: *n* = 60.	**Targeted**: students self-reporting elevated levels of psychological distress.	‘Biodanza’ Dance-based intervention. Intensity not specified. 1 × 90-min group session per week for 4 weeks. Supervised by a trained Biodanza facilitator.	Depressive symptoms (CES-D).	Depressive symptoms were reduced after the Biodanza intervention with a large effect size, Cohen’s d = 1.88, mean diff. 11.78 (SD = 12.67), 95% CI: 7.84 to 15.74. There were no changes in the control group.
						Stress (PSS).	Stress was decreased in the intervention group with a medium effect size, Cohen’s d = 0.79, mean diff. 4.28 (SD = 10.89), 95% CI: 0.89 to 7.68. There were no changes in the control group.
Lubans et al., 2020. [63]	cRCT.	Upper secondary education; secondary school. New South Wales, Australia.	**Total N =** 670. **M age**: 16 (sd = 0.4). **Sex**: M = 55.4%, F = 44.6%. **Waitlist control**: *n* = 333. **Int group**: *n* = 337.	Universal.	‘Burn 2 Learn’ HIIT intervention delivered by teachers during the school day comprised of aerobic and bodyweight resistance movements. 2 × 10-min group sessions per week for 16 weeks, delivered within the education setting. Teachers were trained and supported to deliver the intervention.	Externalizing problems (Strengths and Difficulties Questionnaire)	There were no differences in effects on externalizing problems after the intervention compared to control.
						Internalizing problems (Strengths and Difficulties Questionnaire)	There were no differences in effects on internalising problems after the intervention compared to control. However, there were reductions in internalising problems in two sub cohorts; those who were overweight/obese and those who had elevated mental health issues at baseline.
						Stress (PSS and hair cortisol concentration)	There were no differences in effects on stress measured using the PSS reported between the intervention and control groups at post intervention or 12-month follow up. There were reductions in stress after the intervention for participants who were classified as overweight/obese at baseline. The Burn2Learn intervention had positive effects on hair cortisol concentrations compared to control, adj mean difference: −3.8 (95% CI: −6.67 to 0.93).
						Wellbeing (WEMWBS).	No differences in effects on wellbeing were found between the intervention and control at post intervention or follow up.
Marschin & Herbert, 2021. [49]	Quasi-exp	Post-secondary education; University. Ulm, Germany.	**Total N =** 56. **M age**: 22.39 (sd = 2.02). **Sex**: F = 100%. **Non-PA comparator (expressive writing intervention)**: *n* = 24. **Int group**: *n* = 32.	Universal.	Low intensity aerobic and resistance training intervention delivered at the start of class once weekly. 1 x group session of 5–10 min per week for 6 weeks, delivered by a researcher.	Positive and negative affect (PANAS)	There was a minor decrease in negative affect after both the PA intervention and non-PA comparator groups. There were no effects on positive affect.
						Stress (PSS and SCI)	Stress as measured with the PSS increased for both PA intervention group and control. Stress as measured using the SCI marginally decreased for the PA intervention but increased for the control group.
						Wellbeing (WHOQOL-BREF)	There was no change in psychological subscale score for the PA intervention group while the control group decreased. There was a decrease in physical subscale scores for both intervention and control groups.
Mota et al., 2023 [89]	RCT.	Post-secondary education; University. Alberta, Canada.	**Total N =** 97. **M Age**: 18.25. **Sex**: Male = 28.9%, female = 71.1%. **Intervention group**: *n* = 48. **Control group**: *n* = 49.	Universal.	‘MyViva Plan’ (MVP): a web-based health management programme consisting of 3 pillars; Nutrition, mindfulness and physical fitness; comprised of self-guided health education materials and interactive components. The physical fitness aspect provided participants with self-guided, follow along exercise videos and a scheduling/tracker tool for fitness activities. Participants in the intervention group were instructed to use this platform as frequently as possible for the 12-week intervention period.	Perceived stress (Stress indicator questionnaire).	No significant effects were reported for either intervention or control group on either the total perceived stress score, or any of the subscale scores.
Muir et al., 2020. [79]	Quasi-exp	Post-secondary education; University. Ontario, Canada.	**Total N =** 56. **M age**: 23.08 (sd = 4.9). **Sex**: M = 32.7%, F = 65.3%, Gender invariant = 2%. **(No control group).**	**Targeted**: Participants referred from campus counselling services for seeking help for experiencing anxiety or depression and being sedentary.	‘**UWorkItOutUWin’ programme**: Individualized intervention comprised of one-on-one exercise sessions supervised by a qualified student personal trainer in a private fitness room on campus along with an exercise counselling session each week. 2 × 45 min aerobic and resistance sessions, plus 60-min independent exercise to reach 150 min of moderate to vigorous PA each week, and 1 x exercise counselling session per week for 6 weeks.	Anxiety (MHI-38)	Anxiety was reduced after the exercise intervention, with a mean decrease 4.86 (95% CI, 2.82 to 6.90), with a medium effect size Cohen’s d = 0.68.
						Depressive symptoms (MHI-38)	Depressive symptoms were reduced after the intervention, with a mean decrease 1.31 (95% CI, 0.26 to 2.35), with a small effect size Cohen’s d = 0.36.
						Loss of emotional control (MHI-38).	Although not a significant change, loss of emotional control subscale scores decreased by a mean 2.18 (95% CI, − 0.02 to 4.39).
Nemeroff et al., 2024. [87]	Pilot study.	Post-secondary education; University. New Jersey, USA.	**Total N =** 11. **Age**: 18–20. **Sex**: Male = 9%, female = 91%. No control group.	**Targeted**; students recruited who indicated high levels of stress and anxiety as measured with PSS score indicating moderate or severe perceived stress, and STAI score indicating clinically significant level of anxiety.	Hatha yoga intervention as an alternative to traditional psychotherapy within campus mental health services. Participants received 2 × 60-min hatha yoga classes per week for 6 weeks. Sessions were delivered on campus by two certified yoga instructors.	Stress (PSS); Anxiety (STAI and BAI). Rumination (Rumination-Reflection Questionnaire (RRQ); Pathological worry (Penn state worry questionnaire).	There was a significant reduction in stress after the intervention (*p* = 0.001, ηp² = 0.70). There was a significant reduction in both state (*p* < 0.001, ηp² = 0.77) and trait (*p* < 0.001, ηp² = 0.71) STAI scores, and BAI (*p* = 0.001, ηp²= 0.71) anxiety score after the intervention. There was a significant reduction in participant Pathological worry scores (*p* = 0.001, ηp² = 0.67) between baseline and post-intervention. There was a significant improvement in ruminative thought subscale scores (*p* = 0.007, ηp² = 0.57), however there was no significant change in the reflection subscale (*p* = 0.967).
Newcombe et al., 2024 [67].	Pilot study.	Post-secondary education; University. New Brunswick, Canada.	**Total N =** 16. **M Age**: 21.81. **Sex**: Male = 25%, female = 75%. **Intervention group**: CBT + exercise, *n* = 7. **Control**: Treatment as usual, *n* = 9.	**Targeted;** participants were treatment-seeking university students who met the cut off score for at least mild anxiety, depression or stress on the DASS-21.	CBT plus exercise intervention consisting of 2 x sessions per week for 7 weeks. The first weekly session comprised 45 min of group CBT followed by 30 min of running, with the second weekly session consisting of 30-mins of group running only. CBT sessions were delivered by two mental health professionals, while the exercise component was facilitated by a physical trainer from a local fitness facility. The running sessions gradually increased in intensity, starting with run-walk style sessions to more demanding running later in the intervention. Control group received treatment as usual; they were encouraged to seek traditional counselling/psychotherapy options provided at the campus counselling centre.	Anxiety; Depressive symptoms and Stress (All DASS-21).	There was decreases in scores for Anxiety (−3.69%), depressive symptoms (−18.73%) and stress (−17.90%) for those in the CBT and exercise group, while there were no decrease for the control group. However, significance testing was not completed due to the nature of the pilot study.
Paolucci et al., 2018. [55]	RCT.	Post-secondary education; University. Ontario, Canada.	**Total N =** 61. **M age**: 21 (sd = 2.0). **Sex**: M = 29%, F = 71%. **Non-intervention control group**: *n* = 22. **HIIT group**: *n* = 20. **MCT group**: *n* = 19.	Universal.	**Int 1**: **HIIT aerobic intervention** – high intensity indoor stationary cycling. 3 × 20-min HIIT sessions per week for 6 weeks. **Int 2: Moderate Continuous Training (MCT) aerobic intervention** of indoor stationary cycling. 3 × 27-min moderate intensity sessions per week for 6 weeks. Both were delivered within a laboratory at the University and supervised by researcher(s).	Anxiety (BAI)	Anxiety was decreased after the MCT intervention with a large effect size, Cohen’s d = 0.91. However, anxiety minimally increased following the HIIT intervention.
						Depressive symptoms (BDI)	Both the MCT and HIIT interventions decreased depressive symptoms; with a large effect size for the MCT intervention (Cohen’s d = 0.83) and a medium effect size for the HIIT intervention (Cohen’s d = 0.73).
						Stress (PSS)	Stress was decreased after the MCT intervention with a large effect size, Cohen’s d = 0.91. However, stress increased after the HIIT intervention.
Sabourin et al., 2016. [50]	RCT.	Post-secondary education, University. Canada.	**Total N =** 154. **M age**: 18.8 (sd = 2.2). **Sex**: F = 100%. **Non-PA health education intervention control**: high AS *n* = 37; low AS, *n* = 34. **Int group**: high AS *n* = 44; low AS, *n* = 39.	**Targeted**: participants were female students with either high (*n* = 81) or low (*n* = 73) anxiety sensitivity (AS)	CBT and aerobic exercise – short duration, low intensity running sessions. 3 × 10-min sessions per week for 14 weeks. Running sessions were unsupervised and participants completed them independently.	Anxiety (BAI and DASS-21).	There was no effect on anxiety as measured by both the DASS-21 and BAI after the CBT and running intervention. However, BAI anxiety scores were reduced in the health education control group.
						Anxiety sensitivity (Anxiety Sensitivity Index)	Anxiety sensitivity was reduced in females with high anxiety sensitivity after the CBT and running intervention, however, these reductions did not differ from the health education control group.
						Depressive symptoms (DASS-21)	There was a reduction in depressive symptoms for females with high anxiety sensitivity after both the CBT and running intervention and the health education control intervention; there were no effects in either group for females with low anxiety sensitivity.
						Stress (DASS-21)	Stress decreased after the CBT and running intervention for participants with high anxiety sensitivity. There was no effect on stress for participants with low anxiety sensitivity.
Sharp & Caperchione, 2016. [93]	RCT.	Post-secondary education; University. Canada.	**Total N =** 184. **M age**: 18 (sd = 0.75). **Sex**: M = 47%, F = 53%. **Non-intervention control**: *n* = 89. **Int group**: *n* = 95.	Universal.	Pedometer-based intervention to increase participant’s step count to 10,000 minimum per day. 12 week duration.	Quality of life (SF-12)	There were no differences in effects between the intervention or control on quality of life, with no effect on either the physical health or the mental health subscales. For all participants, the mental health subscale decreased during the intervention period.
						Wellbeing. (GHQ-12)	There was no effect on wellbeing in either intervention or control groups. Wellbeing scores decreased for all participants regardless of group during the intervention period.
Smith et al., 2025 [46].	RCT.	Upper secondary education; secondary school. Newcastle, Australia.	**Total N =** 37. **M Age =** 16.1. **Sex**: male = 40.5%, female = 59.5%. **Int group 1**: Light intensity group, *n* = 12. **Int group 2**: MVPA group, *n* = 12. **Control group**, *n* = 13.	**Universal.**	**Int 1: Light intensity intervention**: 2 × 20-min weekly sessions for 6 weeks consisting of yoga-inspired stretching indoors and leisurely walking outdoors at the school. **Int 2: MVPA intervention**: 2 × 20-min weekly sessions for 6 weeks of group-based interval training delivered indoors, of mixed aerobic and bodyweight resistance-based movements. Sessions were designed to elicit a heart-rate response in the moderate to vigorous range, ≥ 64% max heart rate. Both interventions were delivered by researchers who were physical education qualified.	Psychological distress (Kessler-10); Stress (PSS);	While not significant (*p* > 0.05), there was meaningful improvements in both psychological distress and stress for both the light intensity and MVPA intervention groups compared to the control (cohen’s d’s = −0.38 to −0.54).
Strehli et al., 2023 [90].	Pilot study.	Post-secondary education; University. Utah, USA.	**Total N =** 21. **M Age =** 21. **Sex**: male = 19%, female = 81%. **No control group.**	**Universal.**	Mind-body physical activity intervention consisting of breathing exercises, yoga and walking meditation. The intervention was delivered digitally, unsupervised in which participants followed guided modules for 3 × 10 min sessions per week in their own time, for 8 weeks.	Stress (PSS); Wellbeing (WHO-5);	No significant effects were reported for either stress or wellbeing after the intervention.
Worobetz et al., 2020. [65]	Mixed methods feasibility study.	Post-secondary education; graduate medical school. Limerick, Ireland.	**Total N =** 69. **M age**: 25 (sd = n/a). **Sex**: M = 22%, F = 78%. (no control).	Universal.	‘Med-Well’ mixed PA intervention of supervised, group PA classes including yoga, HIIT, Pilates and aerobic exercise, with health education workshops before sessions. 1 × 60-min group PA class per week for 6 weeks. PA classes were supervised by relevant instructors, and education components were delivered by academic health professionals.	Loneliness & social isolation (UCLA LS-8)	Loneliness and social isolation scores significantly decreased between pre and post intervention.
						Perceived social support (Social Support scale)	There were no effects on perceived social support after the intervention.
						Wellbeing (WHO-5)	There was an increase in wellbeing after the intervention. No effect size reported.
Wierts et al., 2024. [95]	Pilot study.	Post-secondary education; University. Canda.	**Total N =** 72. **M Age =** 20.67. **Sex**: male = 31.94%, female = 63.89%, non-binary = 4.17%. **Intervention group**: walking and running programme, *n* = 36. **Control group**, *n* = 36.	**Targeted;** low active students defined as engaging in less than 150 min of MVPA per week.	8-week running and walking intervention delivered digitally in which participant’s had access to an online platform and divided into groups, in which they individually engaged in weekly running and walking sessions which was shared with their group and contributed to a group challenge, with additional types of activities like weightlifting, cycling or hiking completed in their own time. It also combined other aspects like group support, weekly group virtual coffee chats via Zoom, and maintenance of exercise logs.	Wellbeing (8-item flourishing scale);	There were no significant effects reported for either groups on wellbeing.
Yates et al., 2020. [74]	Mixed methods pilot study.	Post-secondary education; University. USA.	**Total N =** 10. **M age**: 25.94 (6.69). **Sex**: M = 40%, F = 60%. (No control).	**Targeted**: participants were referred from campus mental health services for a diagnosis of depression	Individualized exercise intervention developed for each participant and supervised by a campus wellness coach. Participants self-selected number of sessions (but a mean of 3.3 sessions were recorded per week), for 4 weeks. Participants had the option of using campus facilities but could self-select location.	Depressive symptoms (PHQ-9).	Depressive symptoms were reduced after the intervention, by a mean 3.33 (95% CI: 0.21–6.46), indicative of a large effect size, η²= 0.43.
Zhu et al., 2023. [64]	Pilot study.	Post secondary education; University. USA.	**Total N =** 45. **M age**: 21.93 (sd = 2.4). **Sex**: M = 3.7%, F = 88.88%, other = 7.4%. **Int 1 Peer supported HIIT**: *n* = 14. **Int 2 Self-directed HIIT**: *n* = 13.	**Targeted**: participants recruited for exceeding cut off on a measure indicating elevated depression or anxiety.	**Int 1**: Peer supported HIIT intervention, aerobic bodyweight movements delivered virtually in groups and supervised by researcher(s). **Int 2**: Self-guided, independent HIIT intervention - aerobic bodyweight movements conducted alone independently. Both interventions aimed for total of 150-mins of HIIT per week, through either 2 × 75-mins or 3 × 50 min sessions per week for 8 weeks.	Anxiety (CCAPS-34)	There were beneficial effects on anxiety reported for both the Peer-supported and Self-guided HIIT interventions, with no difference in effects between groups at post intervention. However, peak-to-trough analyses after the intervention at 12-week follow up reported the reductions in anxiety were longer lasting in the self-guided group compared to the peer supported group.
						Depressive symptoms (CCAPS-34)	There was a minimal reduction in depressive symptoms for both intervention groups with no between group differences at post intervention. However, between group differences at 12 week follow up suggested the self-guided intervention had more prominent effects on depression.
Zieff et al., 2022. [56]	Pilot study.	Post-secondary education; University. USA.	**Total N =** 45. **M age**: 20.49 (sd = 2.65). **Sex**: M = 16%, F = 84%. **Non-intervention control**: *n* = 9. **Non-PA comparator (mindfulness intervention)**: *n* = 18. **Int group**: *n* = 18.	**Targeted**: participants exceeded a cut off on a measure indicating high stress.	Meditation and PA intervention of moderate intensity aerobic exercise of indoor stationary cycling or treadmill running combined with guided meditation. 3 × 20-min PA sessions per week, plus encouraged to achieve additional independent exercise to bring total to 150-mins per week, for 4 weeks. Sessions were supervised within the education setting.	Anxiety and Depressive symptoms (DASS-21).	Anxiety and depressive symptoms as measured with the DASS-21 were reduced after the aerobic exercise and meditation intervention with a large effect size, Cohen’s d= −0.97. DASS-21 scores were also reduced after the non-PA comparator (mindfulness-only intervention), also with a large effect size, Cohen’s d= −1.03.
						Stress (PSS)	Stress was reduced after the meditation and aerobic exercise intervention, with a large effect size, Cohen’s d= −1.24. Stress was also reduced in the non-PA comparator (mindfulness-only) intervention, with a large effect size, Cohen’s d= −1.33. The control group also recorded a reduction in stress, a small effect size Cohen’s d= −0.45 was reported.

BAI = Beck Anxiety Inventory; BDI = Beck Depression Inventory; CCAPS-34 = Counselling Centre Assessment of Psychological Symptoms; CD-RISC-10 = Connor–Davidson Resilience Scale; CES-D = Centre for Epidemiological Studies Depression Scale; CY-PSPP = Children and Youth-Physical Self-Perception Profile; DASS-21 = The Depression Anxiety Stress Scales; EDI-2 = Eating Disorder Inventory-2; ERICA = Emotion Regulation Index for Children and Adolescents; HADS = Hospital Anxiety and Depression Scale; HAS = Hamilton Anxiety Scale, HCL-25 = Hopkins Symptom checklist; MBSRQ-AS = Multidimensional Body-Self Relations Questionnaire; MHI-38 = Mental health inventory-38; MSPSS = Multidimensional Scale of Perceived Social Support; QIDS-SR-16 = Quick Inventory of Depressive Symptomatology-self report; PANAS = Positive and Negative Affect Schedule; PHQ-8 = The Stanford Personal Health Questionnaire Depression Scale; PSDQ = Physical Self-Description Questionnaire; SATAQ-4 = Sociocultural Attitudes Toward Appearance Questionnaire-4; STAI = State Trait Anxiety Inventory; SCI = Stress and Coping Inventory; UCLA LS-8 = UCLA loneliness scale; WEMWBS = Warwick-Edinburgh Mental Wellbeing Scale; WHO-5 = World Health Organisation-five item subjective wellbeing Index;. WHOQOL-BREF = World Health Organisation Quality of Life-Brief scale

**Table 3:** Description of areas for future research identified from the results of this review

Research gaps and recommendations for future research		
Category	Summary	Recommendations
**Duration of interventions**	The majority of reported interventions where between 4 and 8 weeks in duration. 11 studies recommend examining interventions of longer durations, as a number of studies reported that durations may be too short to elicit long term mental health benefits.	Future research should examine the effectiveness, feasibility and acceptability of longer-term interventions within the education setting, such as for full semester or full academic year.
**Research with older adolescents**	There was a lack of research within older adolescents of upper secondary education typically aged 16 to 19, as opposed to young adults aged 20 + and typically attending university.	Future research should examine PA interventions within older adolescents within upper-education settings such as high schools, secondary schools and colleges of further education.
**Quality of evidence**	The quality of evidence was mixed, with a comparatively low number of full-scale controlled trials.	There is a need for more well-designed, full scale controlled trials of high quality.
**Use of Technology**	Several studies delivered interventions virtually. With the persistence of remote and virtual working since the Covid-19 pandemic, there is a need to further understanding on virtually delivered PA interventions, which could be effective options to increase flexibility for students.	Future research could examine the effectiveness of virtually delivered interventions, and compare virtually delivered interventions with those delivered in-person.
		Future interventions could incorporate innovative technologies in the delivery of interventions, such as augmented or virtual reality, which have previously shown potential in supporting mental health outcomes [120].
**Inequalities in outcomes**	There was lack of research investigating the differences in outcomes by gender, socioeconomic background (and other demographic factors), and presence of physical or learning disabilities.	There is a need to investigate differences in outcomes of PA interventions, and factors such as differences in preferences, engagement and acceptability of physical activity. More research is needed on the optimal design, feasibility and mental health impacts of physical activity interventions that are accessible to young people with physical or mental disabilities who’s needs may differ to that of the general student population.

## Supplementary Information

Supplementary Material 1.

## Data Availability

No datasets were generated or analysed during the current study.
