# Supplementary material for: A scoping review of physical activity interventions to support the mental health of students aged 16 to 25
Source: Discov Public Health. 2025 Sep 12;22(1):538. doi: 10.1186/s12982-025-00933-8 (PMC12431891; doi:10.1186/s12982-025-00933-8)
Supplement: Supplementary file 1 — Supplementary Material 1. [file 12982_2025_933_MOESM1_ESM.docx]

**Supplementary file 1: Search strategy for Medline database.**

| **Line#** | **Concept 1: Physical Activity** | **Results** |
| --- | --- | --- |
| 1 | exp Exercise/ | 248572 |
| 2 | exp Sports/ | 217662 |
| 3 | Physical Exertion/ | 57519 |
| 4 | exp Physical Fitness/ | 36486 |
| 5 | exp Physical Endurance/ | 37283 |
| 6 | exercise therapy/ or endurance training/ or muscle stretching exercises/ or plyometric exercise/ or resistance training/ | 63140 |
| 7 | exp Exercise Movement Techniques/ | 10378 |
| 8 | (exercis* or sport* or fitness or activit* or athletic* or cardio* or endurance or exertion or movement or training or aerobic* or anaerobic*).tw,kf. | 5554295 |
| 9 | (baseball or basketball or cricket or football or soccer or golf or gymnastic* or hockey or tennis or badminton or rugby or skating or snow sport* or team sport* or volleyball or mountaineer* or skiing or athletic* or yoga or pilates or dance or dancing or walk* or running or marathon runn* or swimming or swim* or nordic walking or stair climbing or jogging or biking or cycling or bicycl* or bike or racing or ballet or zumba or rounders or handball or netball or squash).tw,kf. | 451011 |
| 10 | (calisthenic* or resistance training or weightlifting or "weight lifting" or weight-lifting or isometric* or plyometric* or crossfit or powerlift* or "power lift*" or strength or olympic or "high intensity interval training" or "high-intensity interval training" or HIIT or bodybuild* or warm-up exercise or cool-down exercise or mobility or flexibility or stretching or exercise therap* or remedial exercise* or circuit training or circuit-based exercise or circuit* or parkrun or daily mile).tw,kf. | 856860 |
| 11 | (martial art* or aikido or judo or jujutsu or jujitsu or ju-jitsu or karate or kickboxing or taekwondo or "tae kwon do" or boxing or shadowboxing or boxercise or wrestling or "tai ji").tw,kf. | 5602 |
| 12 | ((moderate or graded or vigorous or intense or low intensity or high intensity or strenuous or light or steady state or long duration or short duration) adj3 (activit* or cardio* or training or exercis*)).tw,kf. | 77084 |
| 13 | ((activ* or physical or exerci*) adj3 (play* or lesson* or transport* or travel* or commut* or recess* or class* or extracurricular or extra-curricular)).tw,kf. | 97870 |
| 14 | ((gym or gymnasium or school gym or (fitness or leisure or wellness or exercise or activity or recreation*)) adj2 (cent* or club* or class* or facilit*)).tw,kf. | 18332 |
| 15 | ("PE lesson" or Physical Education or "PE" or gym class* or exercise class*).tw,kf. | 71024 |
| 16 | OR 1-15 | 6535004 |
|  | **Concept 2: Mental Health** |  |
| 17 | Mental Health/ | 62658 |
| 18 | exp Anxiety Disorders/ | 91003 |
| 19 | exp Mood Disorders/ | 170570 |
| 20 | Anxiety/ | 107919 |
| 21 | Psychological Well-Being/ | 257 |
| 22 | Depression/ | 152106 |
| 23 | Resilience, Psychological/ | 8424 |
| 24 | Psychological Distress/ | 4200 |
| 25 | Adolescent Psychiatry/ | 3097 |
| 26 | self concept/ or body image/ or self efficacy/ or self-compassion/ | 99222 |
| 27 | ((Mental or emotion* or psychological or psychiatric) adj3 (health or illness* or wellbeing or well-being or "well being" or illhealth or ill-health or "Ill health" or "ill being" or illbeing or ill-being or problem* or issue* or difficult* or condition* or disorder* or disease* or symptom* or distress*)).tw,kf. | 500099 |
| 28 | (depress* or anxiety or anxious or dysthymi* or mood* or resilien* or wellbeing or well-being or "well being" or "quality of life" or mood disorder* or affective disorder* or isolation or loneliness or psychosocial).tw,kf. | 1562182 |
| 29 | (suicid* or "self harm" or self-harm or self-injury or "self-injurious behavior").tw,kf. | 101965 |
| 30 | (Self adj3 (esteem or efficacy or concept or image or worth or confidence)).tw,kf. | 83660 |
| 31 | ((External$ing or internali$ing) adj3 (symptom* or disorder* or behavior* or behaviour* or problem* or condition*)).tw,kf. | 0 |
| 32 | OR 17-31 | 2067833 |
|  | **Concept 3: Students/Education Settings** |  |
| 33 | Students/ | 81151 |
| 34 | exp Schools/ | 146912 |
| 35 | School Mental Health Services/ | 61 |
| 36 | Student Health Services/ | 3198 |
| 37 | (student* or pupil* or schoolchild*).tw,kf. | 405165 |
| 38 | ((secondary or upper-secondary or "upper secondary" or "senior secondary") adj3 (education or school* or college* or institut* or academ* or student* or pupil* or study or studies)).tw,kf. | 34066 |
| 39 | (further education or "FE" or 6th-form or "6th form" or "sixth form" or sixth-form).tw,kf. | 124979 |
| 40 | (high-school* or "High school*" or "community college").tw,kf. | 41103 |
| 41 | (Universit* or college*).tw,kf. | 587878 |
| 42 | ((Universit* or College* or vocation* or technical or higher or third-level or "third level" or "3rd level" or 3rd-level or Post-secondary or undergraduat* or postgraduat* or "post secondary" or tertiary) adj3 (student* or pupil* or education or college* or institut* or academ*)).tw,kf. | 263712 |
| 43 | ((education or school* or (education* or school* or academic)) adj3 (setting* or environment*)).tw,kf. | 37464 |
| 44 | OR 33-43 | 1176363 |
|  | **Concept 4: Age Range 16 - 24** |  |
| 45 | Adolescent/ | 2221815 |
| 46 | Young Adult/ | 1014632 |
| 47 | (adolescen* or young adult* or teen* or young people or child* or youth or young person).tw,kf. | 2005743 |
| 48 | OR 45-47 | 3944503 |
|  | **Study Design** |  |
| 49 | Randomized Controlled Trial.pt. | 600219 |
| 50 | Random Allocation/ | 106968 |
| 51 | Controlled Clinical Trial.pt. | 95422 |
| 52 | (randomi#ed or randomi#ation or randomi#ing).tw,kf. | 809137 |
| 53 | (RCT or cRCT or "at random" or (random* adj3 (administ* or allocat* or assign* or class* or cluster or crossover or cross-over or control* or determine* or divide* or division or distribut* or expose* or fashion or group* or number* or place* or pragmatic or quasi or recruit* or split or substitut* or treat*))).tw,kf. | 754388 |
| 54 | trial.ti. | 288679 |
| 55 | ((control* or compar*) adj4 group*).ab. | 1066797 |
| 56 | ((control* or compar*) and ((usual adj2 (activit* or practice?)) or "as usual")).ab. | 60887 |
| 57 | (control* and (trial or study)).tw,kf. | 2323763 |
| 58 | Feasibility Studies/ | 83747 |
| 59 | Pilot Projects/ | 148675 |
| 60 | ((feasibilit* or pilot) adj (study or project? or program*)).tw,kf. | 144128 |
| 61 | quasi*.tw,kf. | 84474 |
| 62 | factorial.tw,kf. | 37358 |
| 63 | attention-control.tw,kf. | 2088 |
| 64 | Controlled Before-After Studies/ | 734 |
| 65 | controlled before and after.tw,kf. | 1318 |
| 66 | Interrupted Time Series Analysis/ | 1903 |
| 67 | (time series or time point? or repeat* measur*).tw,kf. | 248819 |
| 68 | (pre-intervention? or preintervention? or "pre intervention?" or post-intervention? or postintervention? or "post intervention?").tw,kf. | 37672 |
| 69 | ((before adj5 after) or pre-post or (pre adj5 post) or ((pretest or "pre test") and (posttest or "post test"))).tw,kf. | 569042 |
| 70 | (control* and (before adj10 (after or during))).tw,kf. | 169858 |
| 71 | (pre-study or pre-program* or pre-project? or pre-campaign? or pre-initiative? or pre-mandat* or pre-strateg* or post-study or post-program* or post-project? or post-campaign? or post-initiative? or post-mandat* or post-strateg* or ((before or after) adj3 (pilot or program* or project? or campaign? or initiative? or mandat* or strateg*))).tw,kf. | 36896 |
| 72 | OR 49-71 | 4402943 |
| 73 | 16 and 32 and 44 and 48 and 72 | 5839 |
|  | **Limits: 2015 – current, English Language** |  |
| 74 | limit 73 to (yr="2015 -Current" and english) | 3545 |
